# Supplementary material for: Alpha-ketoglutarate mitigates insulin resistance and metabolic inflexibility in a mouse model of Ataxia-Telangiectasia
Source: Nat Commun. 2025 Oct 21;16:9312. doi: 10.1038/s41467-025-64360-8 (PMC12540671; doi:10.1038/s41467-025-64360-8)
Supplement: Supplementary file 1 — Supplementary Information [file 41467_2025_64360_MOESM1_ESM.pdf]

Supplementary Information

Alpha-ketoglutarate mitigates insulin resistance and metabolic inflexibility in a mouse model of Ataxia-Telangiectasia

Jacquelyne Ka-Li SUN[1], Ronald P. HART[2], Karl HERRUP[3], Amy Zexuan PENG[1], Genper Chi-Ngai WONG[1], Deng WU[1], Kin-Ming KWAN[1,4,5], Kim Hei-Man CHOW[1,6,#]

[1]School of Life Sciences, Faculty of Science, The Chinese University of Hong Kong, Hong Kong

[2]Department of Cell Biology and Neuroscience, Rutgers University, Piscataway, New Jersey, USA

[3]Department of Neurobiology, School of Medicine, University of Pittsburgh, Pittsburgh, Pennsylvania, USA.

[4]State Key Laboratory of Agrobiotechnology, The Chinese University of Hong Kong, Hong Kong

[5]Centre of Cell and Developmental Biology, The Chinese University of Hong Kong, Hong Kong

[6]Gerald Choa Neuroscience Institute, The Chinese University of Hong Kong, Hong Kong

# To whom correspondence should be addressed:
Kim Hei-Man CHOW
Email: heimanchow@cuhk.edu.hk
Phone: (+852)3943-1530

Table of Contents

Supplementary Fig.1. **Supplementary data reinforcing the comprehensive metabolic profiling in ATM deficiency**

Supplementary Fig.2. **Additional data corroborating insulin-activated ATM modulates key regulators of aerobic glycolysis in the cerebellum.**

Supplementary Fig.3. Supplementary details for phosphoproteome analyses.

Supplementary Fig.4. Supplementary details of total proteome analyses.

Supplementary Fig.5. Supplementary evidence corroborating ATM deficiency disrupts glycolytic capacity and reprograms to glutamine dependence in the cerebellum.

Supplementary Fig.6. **PKM2 interacts c-Myc mRNA to enhance its protein translation in fibroblasts harvested from individuals with A-T.**

Supplementary Fig.7. **Glutaminase-2 is a downstream target of c-Myc.**

Supplementary Fig.8. **Vulnerability of Purkinje cells to cell death in the absence of ATM.**

Supplementary Fig.9. Representative immunohistology images of sagittal sections dissecting the cerebellar vermis, demonstrating irregularities in ALDOC+ Purkinje cell densities across different lobes in *Atm*-KO (n=8).

Supplementary Fig.10. Representative immunohistology images of sagittal sections dissecting the cerebellar vermis, **illustrating intricate alterations in IRS1 signaling within Purkinje cells across various lobes in *Atm*-KO.**

Supplementary Fig.11. Investigation of ALDOC-associated dysregulation and alterations in ATM deficiency.

Supplementary Fig.12. *ALDOC* is a potential target gene regulated by HIF1α.

Supplementary Fig.13. Supplementary data corroborating comprehensive metabolic profiling in the context of hyperinsulinemia.

Supplementary Fig.14. Promoter analysis of glycolytic genes (Fig.5j) for HIF1α binding using the ChIP-Atlas database.

Supplementary Fig.15. Analysis of gene set enrichment using GSEA for markedly altered genes in human cerebellar samples from A-T patients (GSE61019).

Supplementary Fig.16. Supplementary information regarding indirect calorimetry results from mice subjected to the CaAKG treatment regimen.

Supplementary Fig.17. Representative immunohistology images of sagittal sections dissecting the cerebellar vermis illustrating changes in the number of IP3R1+ Purkinje cells across various lobes in *Atm*-KO mice.

Supplementary Fig.18. Representative immunohistology images of sagittal sections dissecting the cerebellar vermis illustrating changes in the network of VGlut2 climbing fibers across various lobes in *Atm*-KO mice.

Supplementary Table 1. Special reagents list.

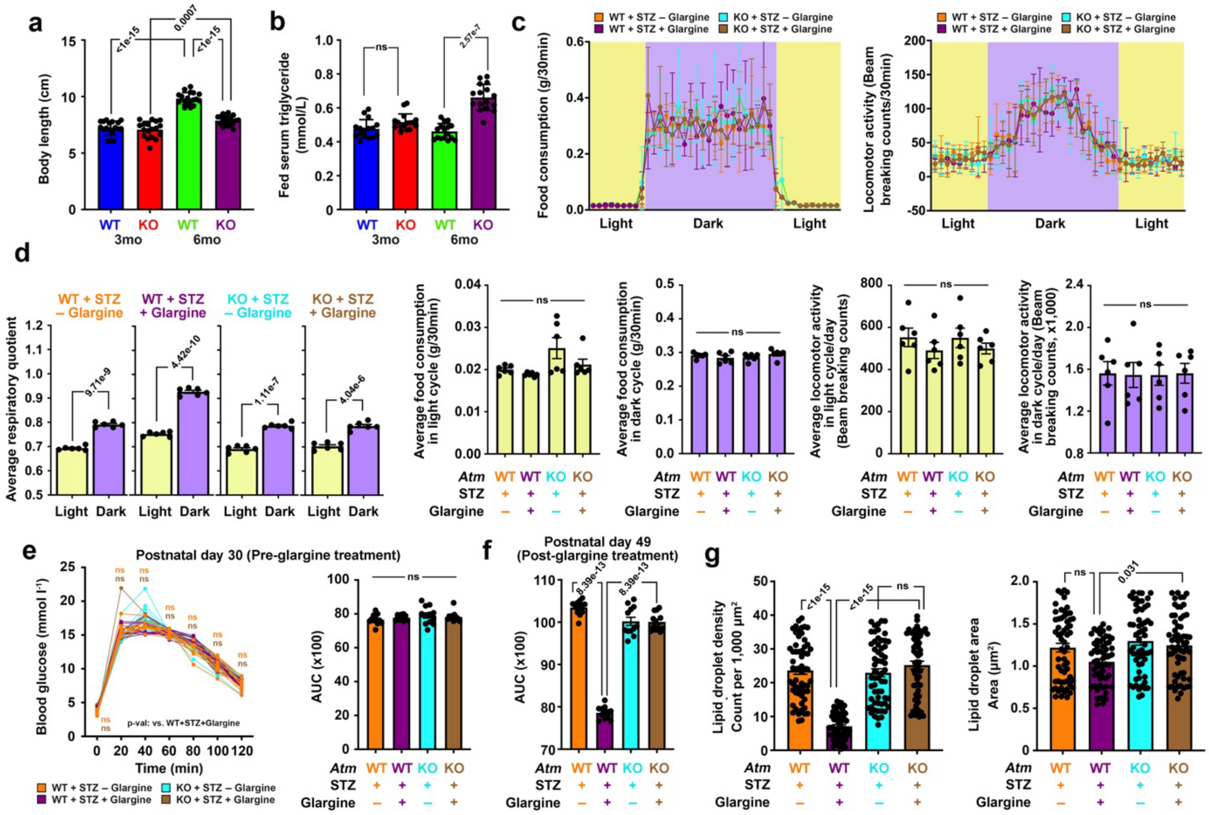

Supplementary Fig.1. Supplementary data reinforcing the comprehensive metabolic profiling in ATM deficiency. a, Variations in body length (n=18, one-way ANOVA). **b**, Overview of serum triglyceride levels in the fed state (n=16, Kruskal-Wallis test). **c,** Temporal variations in indirect calorimetry assessments are depicted. The data is illustrated across light (yellow) and dark (purple) cycles spanning a 24-hour long of measurement. Alterations in patterns of both food intake and locomotor activity are delineated (n=6). **d,** Summary of changes in RQs (Corresponds to Fig.1j; n=6, two-tailed unpaired t-test), food consumption (Corresponds to Supplementary Fig.1c; n=6, Kruskal-Wallis test for light cycle, one-way ANOVA for dark cycle) and locomotor activities (Corresponds to Supplementary Fig.1c; n=6, one-way ANOVA) in light versus dark cycle. **e,** Baseline IGTT outcomes prior to intervention (n=12, two-way ANOVA). Summary of the Area Under Curves (AUCs) is presented on the right (n=12, Kruskal-Wallis test). **f,** Summary of area under curves (AUCs) of post-treatment IGTT (Corresponds to Fig.1k) (n=12, one-way ANOVA). **g,** Alterations in lipid droplet density and lipid droplet area (n=60, Kruskal-Wallis test). N presents biological replicates. Values represent the mean ± s.d. Source data are provided as a Source Data file.

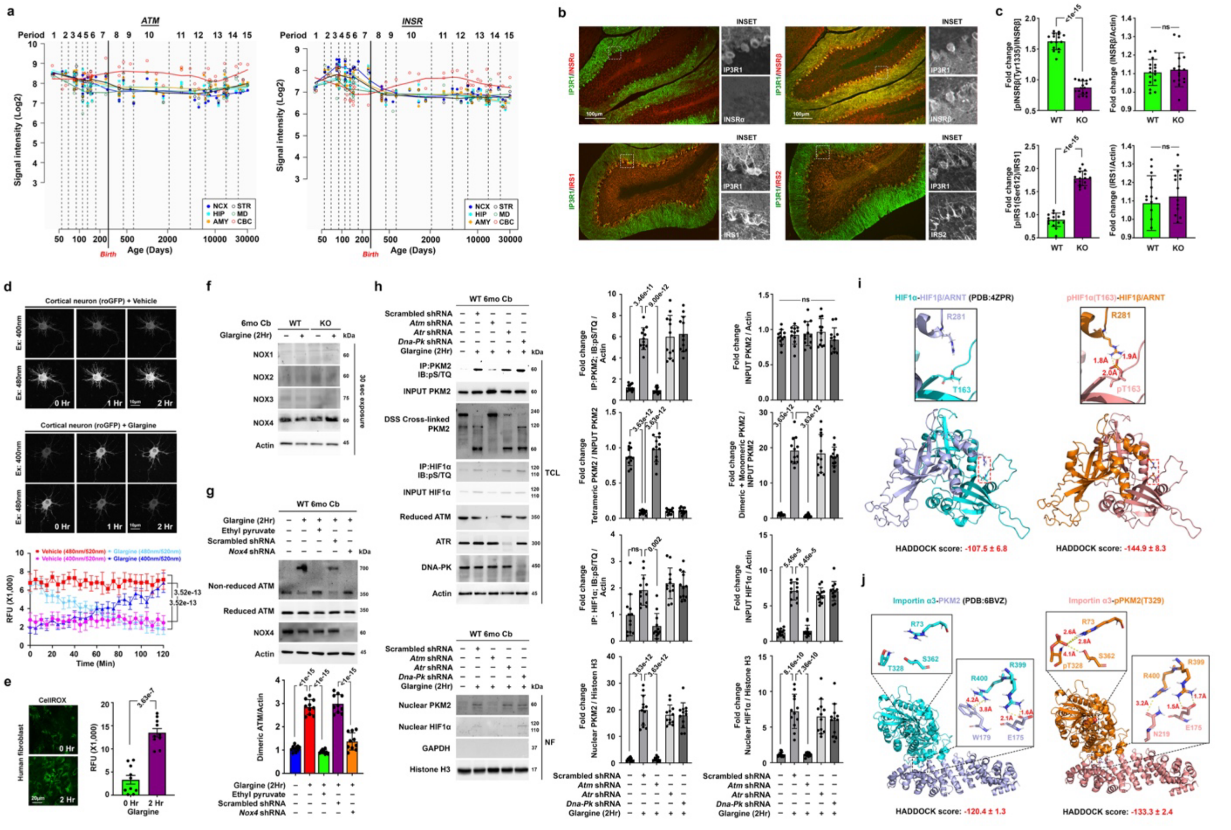

Supplementary Fig.2. Additional data corroborating insulin-activated ATM modulates key regulators of aerobic glycolysis in the cerebellum. a, *ATM* and *INSR* exon array signal intensity (n=5 male brains per period) extracted from the Human Brain Transcriptome database[25]. **b,** Representative images of sagittal sections traversing the cerebellar vermis, demonstrating the distribution of essential insulin signaling elements (n=6). **c,** Quantification of relative immunoblot intensities of blots presented in Fig.2d (n=16, two-tailed unpaired t-test). **d,** Illustrative time-lapse images displaying signals of roGFP biosensors in primary cortical neurons following treatment with either vehicle or glargine. Quantitative analysis of signals captured across various excitatory wavelengths is presented (n=10, two-way ANOVA). **e,** Representative CellROX images of human fibroblast exposed to glargine for 2 hours (n=10, two-tailed unpaired t-test). **f,** Representative immunoblots depicting the NOX family of proteins in lysates obtained from acute *ex vivo* cerebellar tissue cultures exposed to 2 hours of glargine (n=6). **g,** Representative immunoblots depicting the effects of NOX4 knockdown and hydrogen peroxide (H2O2) scavenging by ethyl pyruvate on ATM dimerization (n=10, one-way ANOVA). **h,** Representative immunoblots illustrating the essential function of ATM but no other key members of the PI3K-like protein kinase family (including ATR and DNA-PK), in facilitating the phosphorylation, interaction, and nuclear translocation of PKM2 and HIF1α. Quantifications of the relative intensities of immunoblot bands are displayed on the right (n=12, one-way ANOVA for all except for the quantifications of "IP:HIF1α; IB: pS/TQ/ Actin" and "INPUT HIF1α /Actin" where Kruskal-Wallis test was used). **i, j,** Molecular models of **i,** HIF1α-HIF1β (ARNT) (PDB: 4ZPR) and **j,** importin α3-PKM2 (PDB: 6BVZ) showing how ATM-mediated phosphorylation at residue T163 on HIF1α and at residue T329 on PKM2 facilitate protein-protein interactions in terms of HADDOCK scores. Key interacting residues and the distance of interactions on their

corresponding binding partners are shown. Unless otherwise specified, all *ex vivo* glargine treatments were performed in 100 nM for 2 hours. N presents biological replicates. Values represent the mean ± s.d. Source data are provided as a Source Data file.

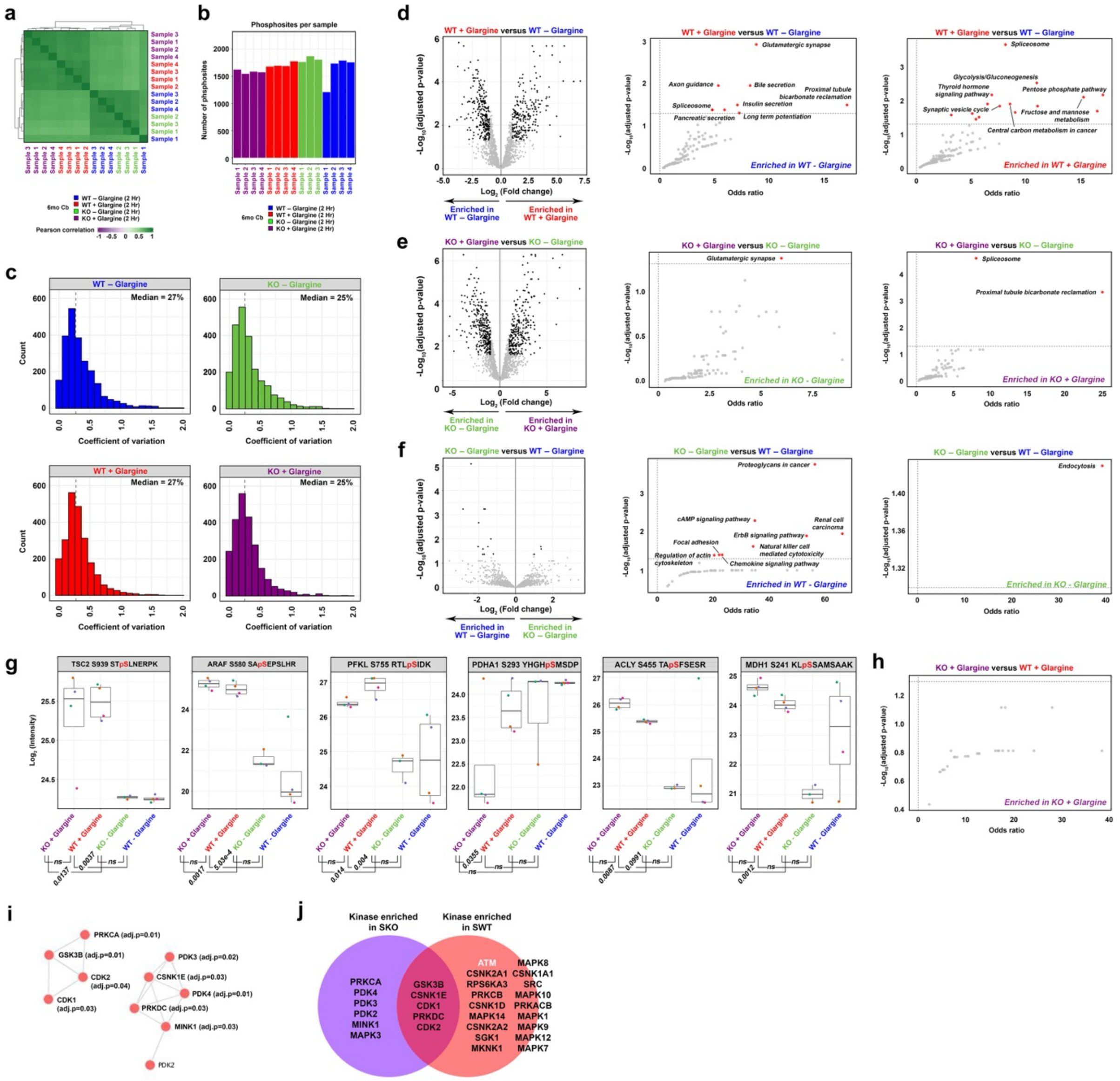

Supplementary Fig.3. Supplementary details for phosphoproteome analyses. a, Correlation plot visualizes the relationship among different samples. The darker the stronger relevance between each sample (n=4 for all except n=3 for KO-Glargine group). **b,** Bar chart revealing the phosphosites quantified per sample after pre-processing (n=4 for all except n=3 for KO-Glargine group). **c,** Histogram plots revealing the sample coefficient of variation, which illustrates the degree of variation relative to the overall mean (n=4 for all except n=3 for KO-Glargine group). **d-f,** Volcano plot illustrates statistical significance (adjusted p-value) versus change magnitude (fold change) in phosphopeptides detected in **d,** "WT+Glargine" versus "WT-Glargine"; **e,** "KO+Glargine" versus "KO–Glargine"; or **f,** "KO–glargine" versus "WT – Glargine" groups of samples. Significance is conferred upon hits with an adjusted p-value of 0.05, utilizing the Benjamini-Hochberg method alongside a |log2 fold change| of 1 (Limma with Benjamini-Hochberg correction). Functional pathway enrichment analyses of phosphopeptides with reference to the KEGG database were conducted on the Enrichr platform[175] (n=4 for all except n=3 for KO-

Glargine group, Fisher exact test with correction). **g,** Relative intensities of TSC2 (S939), ARAF (S580), PFKL (S755), PDHA1 (S293), ACLY (S455) and MDH1 (S241) phosphopeptide detected in different treatment groups (n=4 for all except n=3 for KO-Glargine group, one-way ANOVA**).** **h,** Functional pathway enrichment analysis and **i,** kinase enrichment analysis of phosphopeptides enriched in “KO + Glargine” group with reference to the KEGG database and kinase enrichment database on the Enrichr platform[13] (Fisher exact test with correction), respectively. j, Venn diagram showing the difference and overlapping identities of kinase identified from the kinase enrichment analysis of phosphopeptides identified from both “WT+Glargine” and “KO+Glargine” groups. N presents biological replicates. Values represent the mean ± s.d. Source data are provided as a Source Data file.

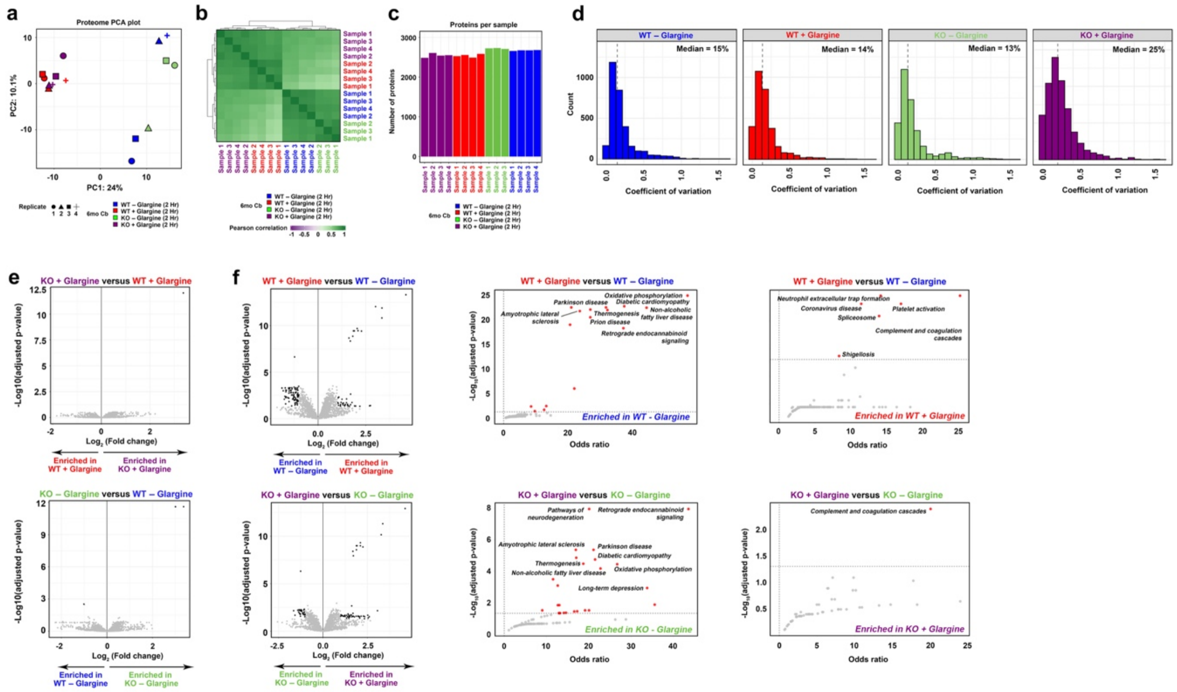

Supplementary Fig.4. Supplementary details of total proteome analyses. a, PCA plot of all samples in different treatment groups based on their total proteome to emphasize the variations (n=4 for all except n=3 for KO-Glargine group). **b,** Correlation plot visualizes the relationship among different samples. The darker the stronger relevance between each sample (n=4 for all except n=3 for KO-Glargine group). **c,** Bar chart revealing the number of peptides quantified per sample after pre-processing (n=4 for all except n=3 for KO-Glargine group). **d,** Histogram plots revealing the sample coefficient of variation, which illustrates the degree of variation relative to the overall mean (n=4 for all except n=3 for KO-Glargine group). **e-f,** Volcano plots illustrate statistical significance (adjusted p-value) versus change magnitude (fold change) in total peptides detected in **e,** "KO+Glargine" versus "WT+Glargine" and "KO–Glargine" versus "WT–Glargine"; so as **f,** "WT+Glargine" versus "WT–Glargine" and "KO + glargine" versus "KO – Glargine" groups (n=4 for all except n=3 for KO-Glargine group). Significance is conferred upon hits with an adjusted p-value of 0.05, utilizing the Benjamini-Hochberg method alongside a |log2 fold change| of 1. Functional pathway enrichment analyses of peptides with reference to the KEGG database were conducted on the Enrichr platform[175] (Fisher exact test with correction). N presents biological replicates. Values represent the mean ± s.d. Source data are provided as a Source Data file.

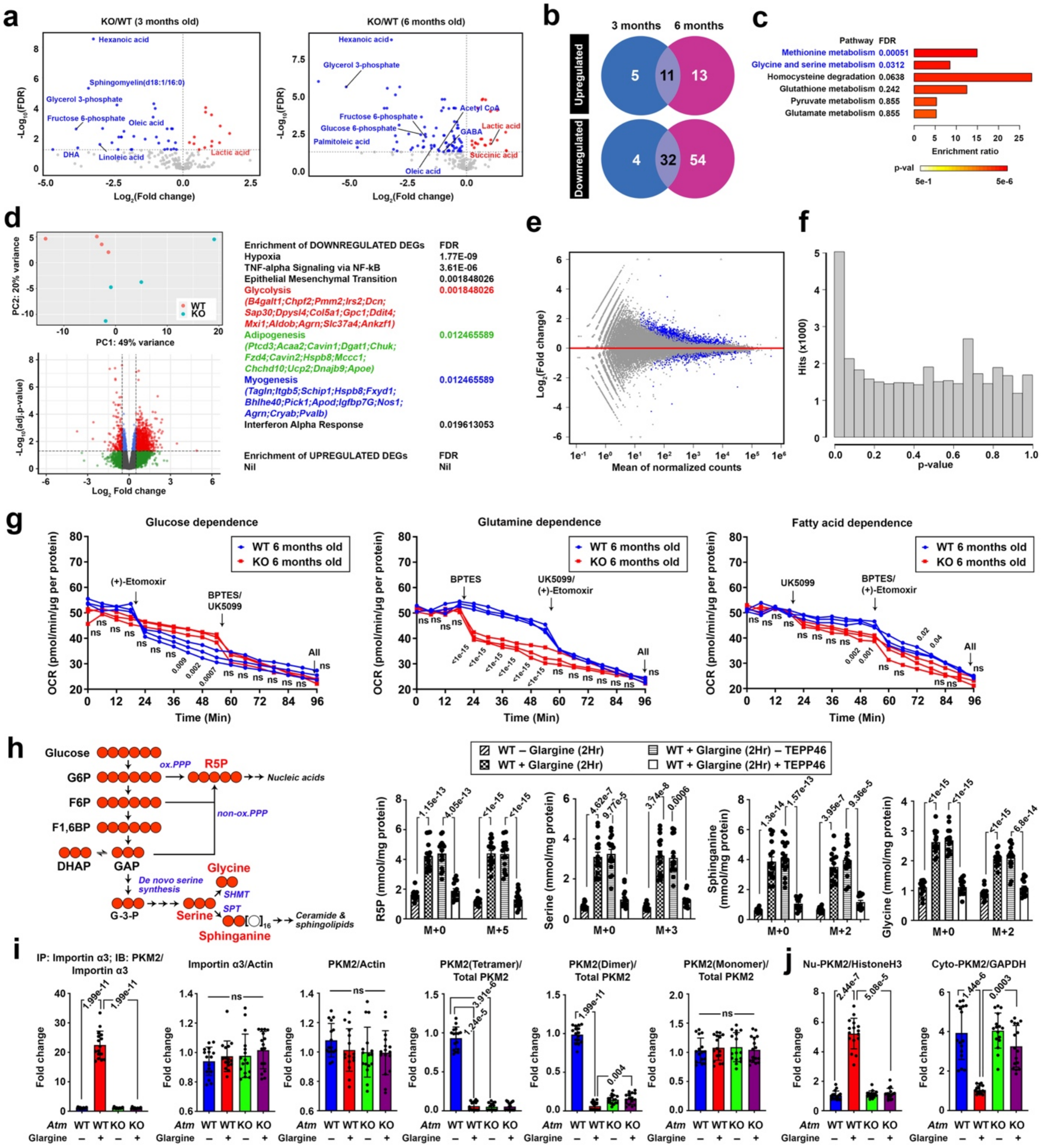

Supplementary Fig.5. Supplementary evidence corroborating ATM deficiency disrupts glycolytic capacity and reprograms to glutamine dependence in the cerebellum. a, Volcano plots illustrate the metabolites that are up- and down-regulated in cerebellar tissues collected from 3-month-old (Left) and 6-month-old mice (n=6, Unpaired multiple t-test corrected by Benjamini, Krieger, and Yekutieli procedure). **b,** Venn diagram illustrates the numbers of shared up- and down-regulated metabolites identified across both age groups. **c,** Metabolite set enrichment analysis of the metabolites commonly upregulated in cerebellar tissues of *Atm*-KO mice aged 3 and 6 months, as determined from comprehensive global metabolite profiling mentioned in panels **a,** and **b,** (Globaltest with Bonferroni corrections). **d,** Upper left: Principal component analysis

(PCA) plot revealed distinct clustering of samples from the *Atm*-WT and KO groups (n=4). Lower left: Volcano plot indicated 1,003 transcripts exhibited significant differences between the two groups (Adjusted p-value<0.05; Log2(Fold change)>|0.5|) (Limma with Benjamini-Hochberg correction). Right: Pathway network analysis of significantly upregulated and downregulated genes conducted with Enrichr (Fisher exact test with correction). **e,f,** Adequate quality assurance of the findings within the bulk transcriptome dataset was affirmed through the **e,** mean difference plot and **f,** adjusted p-value histogram. **g,** Illustrative oximetric traces were utilized to evaluate the impact of glucose, glutamine, and fatty acids on the basal oxygen consumption rate (OCR) (n=3, two-way ANOVA). **h,** Left: schematics of $^{13}C_6$-U-glucose carbon flow. Right: Mass isotopologue analysis conducted on ribulose-5-phosphate (R5P), serine, sphinganine, and glycine in acute *ex vivo* cerebellar cultures (n=16, one-way ANOVA for all except for "M+0, M+3 Serine" and "M+2 Sphinganine" where Krustal-Wallis test was used). **i,** Measurement of the relative immunoblot intensities depicted in Fig.3f (n=16, one-way ANOVA for all except for "Importin-α3/Actin" and "PKM2 (Tetramer)/PKM2" where Kruskal-Wallis test was used). **j,** Measurement of the relative immunoblot intensities depicted in Fig.3g (n=16, Kruskal-Wallis test). Unless otherwise specified, all *ex vivo* glargine and TEPP-46 treatments were performed in 100 nM for 2 hours. N presents biological replicates. Values represent the mean ± s.d. Source data are provided as a Source Data file.

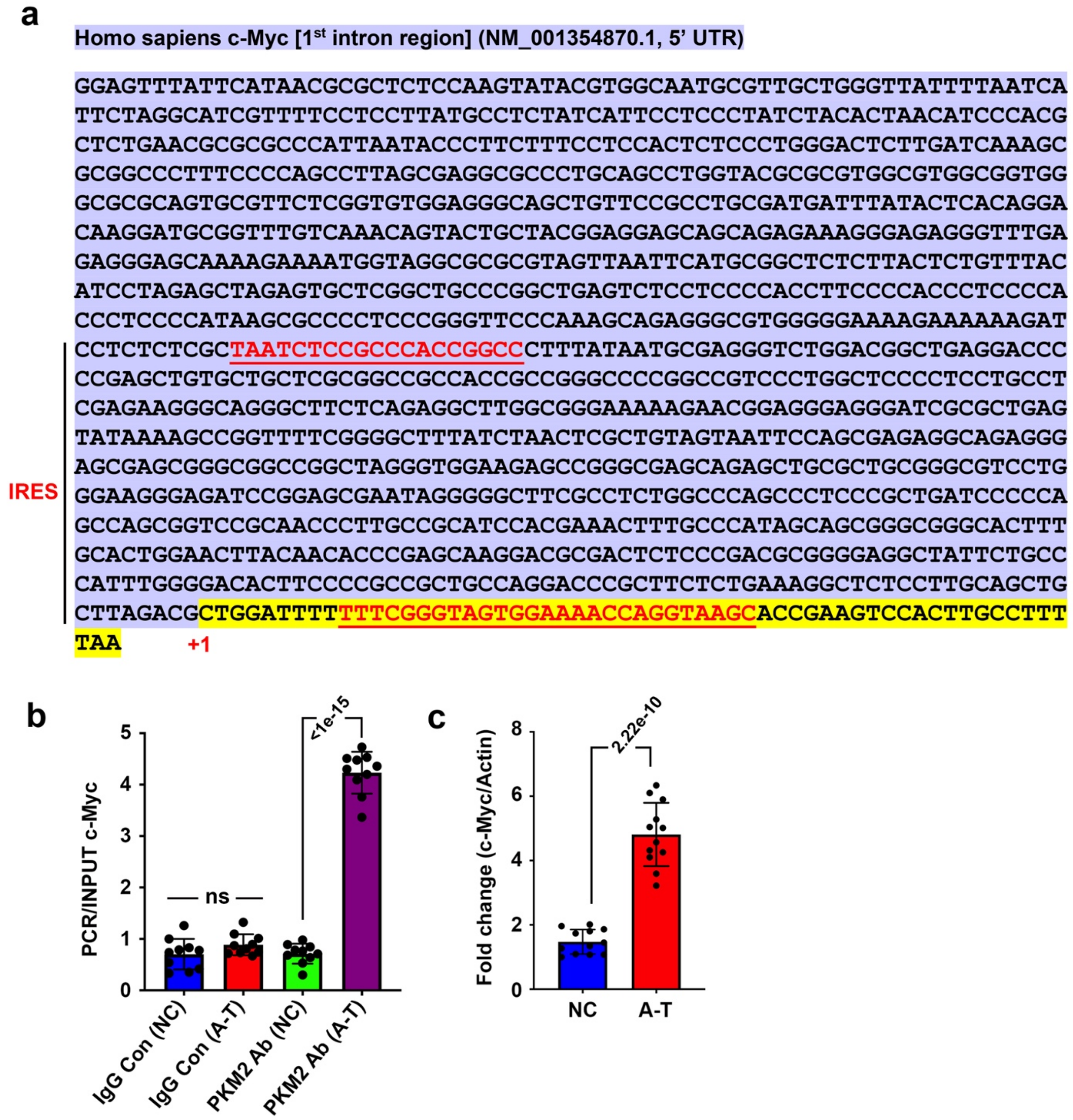

Supplementary Fig.6. PKM2 interacts c-Myc mRNA to enhance its protein translation in fibroblasts harvested from individuals with A-T. a, Sequence within the c-Myc messenger RNA that functions as an internal ribosome entry site (IRES). **b**, Quantification of DNA blots presented in Fig.3i (n = 10; one-way ANOVA). **c,** Quantification of immunoblots presented in in Fig.3j (n =12, two-tailed unpaired t-test). N presents biological replicates. Values represent the mean ± s.d. Source data are provided as a Source Data file.

a

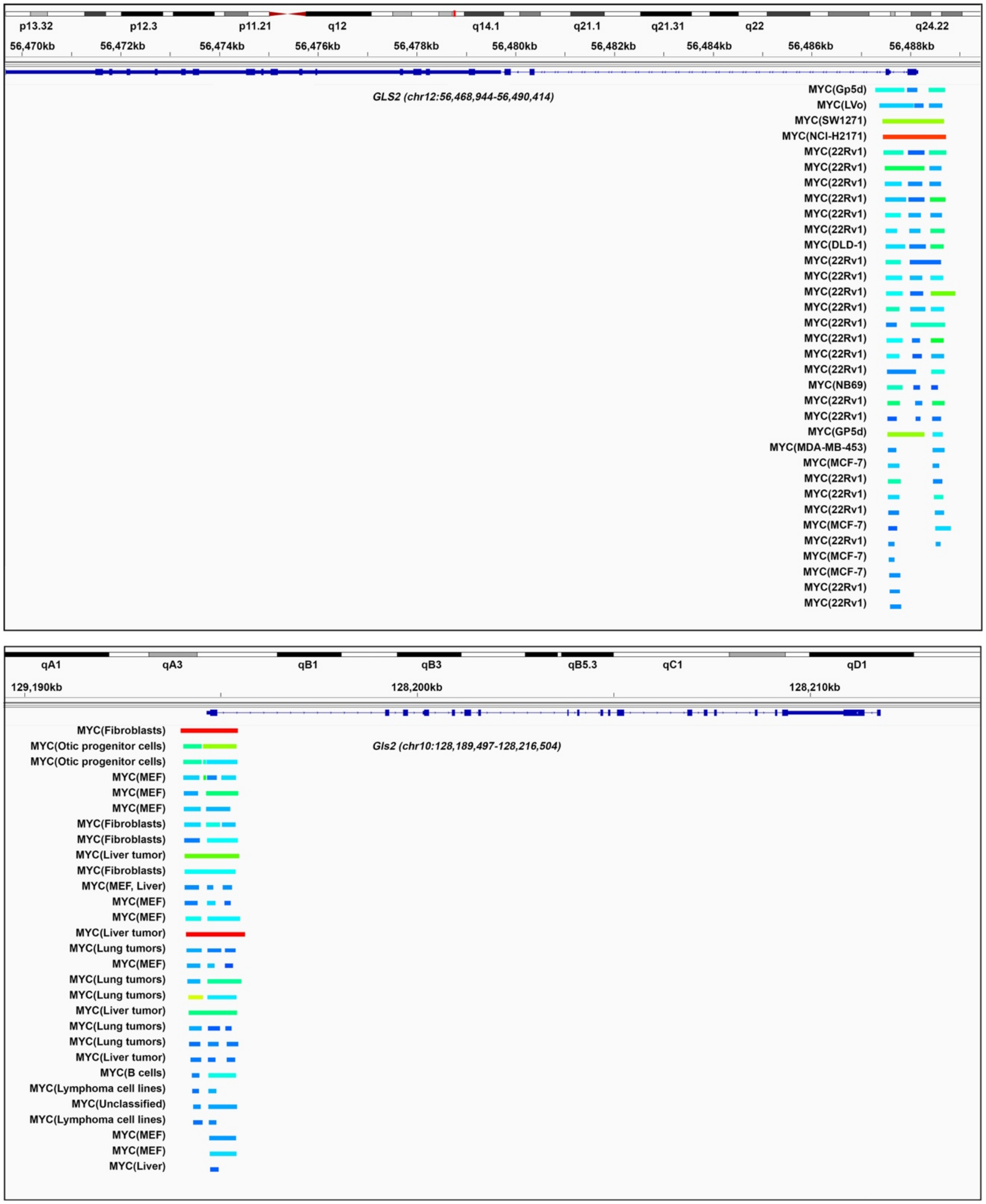

b

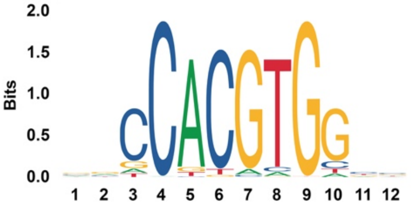

c

Human *GLS2* promoter

Predicted sequence	Start	End	Strand	Score
GGCCATGTGGAT	-1401	-1930	+	9.42008
CACCGCGTGGTC	-2137	-2126	+	8.908994

Mouse *Gls2* promoter

Predicted sequence	Start	End	Strand	Score
CGCACCTGGC	-674	-665	+	9.968428
TCCACATGGC	-1314	-1323	–	9.0619
AGCACGTGAC	-1120	-1111	+	9.012213
CCCAGGTGGC	-695	-686	+	8.90826
GGCACGGGGC	-157	-166	–	8.251954

d

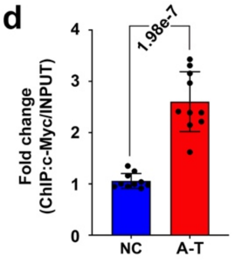

Supplementary Fig. 7. Glutaminase-2 is a downstream target of c-Myc. a, Investigation of the human and mouse glutaminase-2 (*GLS2/Gls2*) gene promoter for c-Myc binding through ChIP-Atlas analysis. **b**, Diagram illustrating the human c-Myc consensus binding motif obtained from the JASPAR database. **c,** Anticipated c-Myc binding motifs within the promoter region of the human and mouse glutaminase-2 (*GLS2/Gls2*) gene. The consensus site is highlighted in red. **d,** Quantification of DNA blots presented in Fig.3k (n=10, two-tailed unpaired t-test). N presents biological replicates. Values represent the mean ± s.d. Source data are provided as a Source Data file.

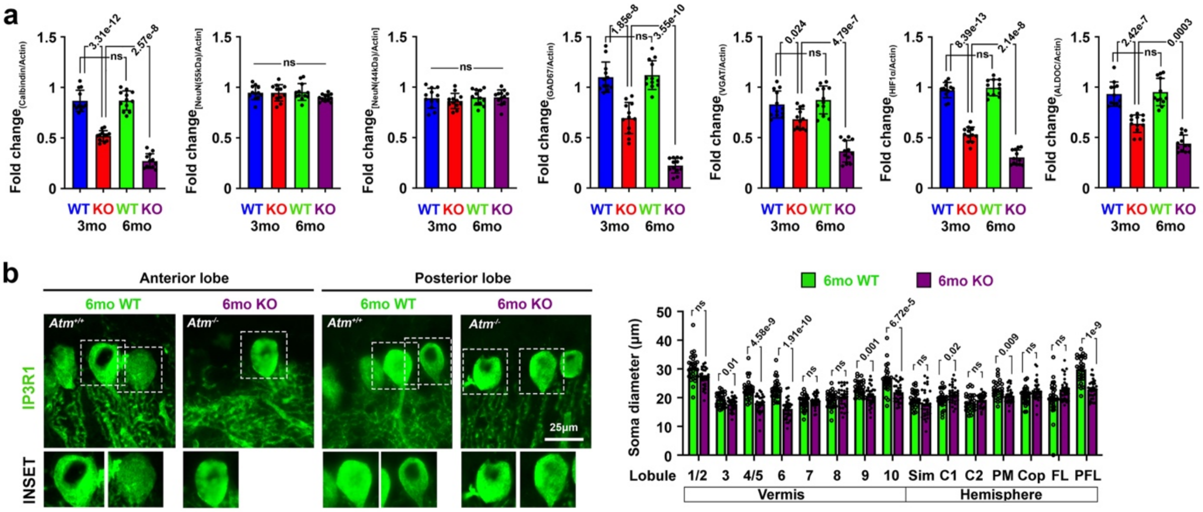

Supplementary Fig. 8. Vulnerability of Purkinje cells to cell death in the absence of ATM. a, Quantification of the immunoblot intensities depicted in Fig.4b (n=12, one-way ANOVA). **b,** Representative images illustrate alterations in Purkinje cell (IP3R1+) soma size (mean diameter). Measurement of the Purkinje cell soma diameter across different cerebellar regions is shown in the right (n=30, two-tailed unpaired t-test). N presents biological replicates. Values represent the mean ± s.d. Source data are provided as a Source Data file.

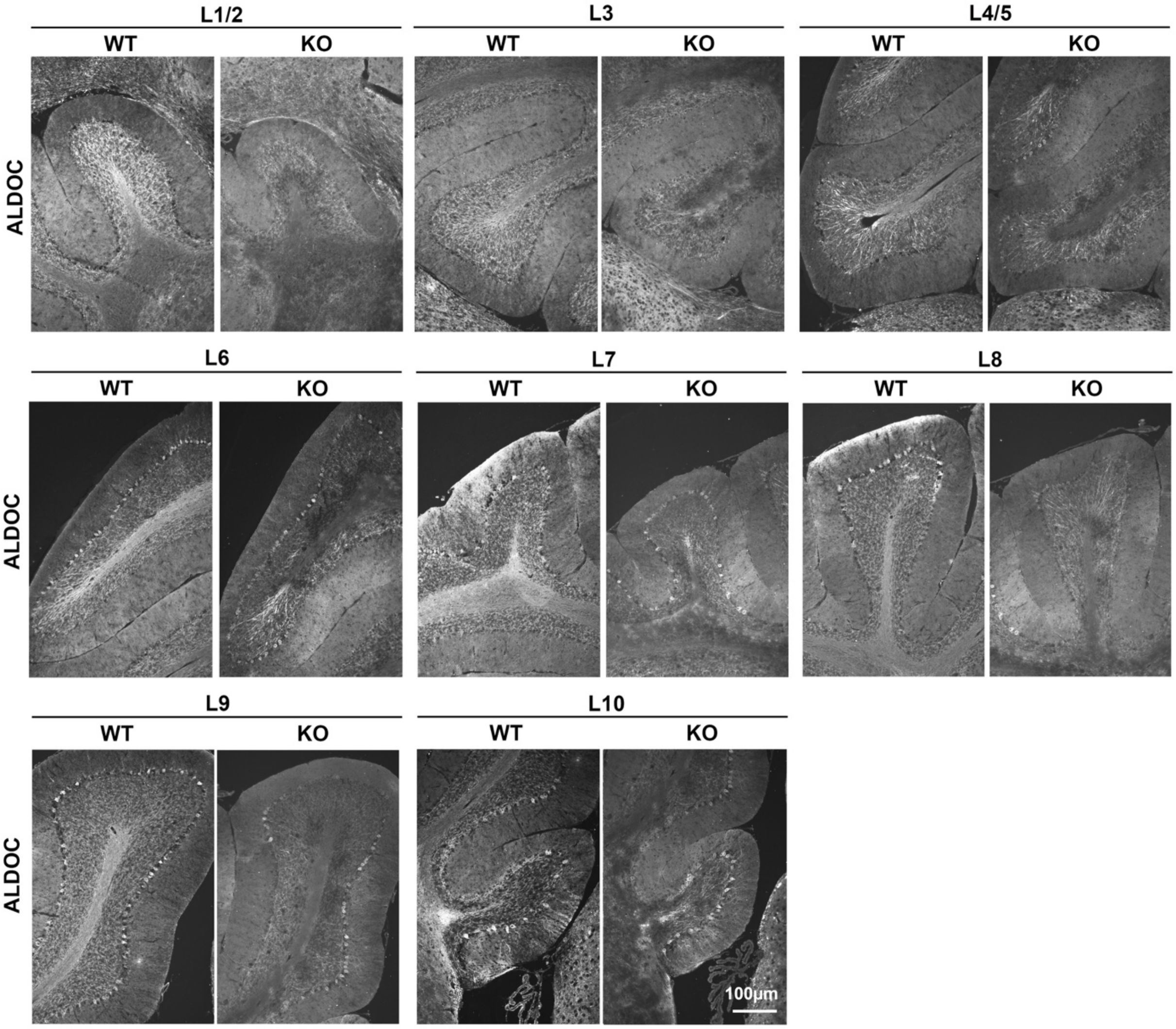

Supplementary Fig.9. Representative immunohistology images of sagittal sections dissecting the cerebellar vermis, demonstrating irregularities in ALDOC+ Purkinje cell densities across different lobes in *Atm*-KO (n=8).

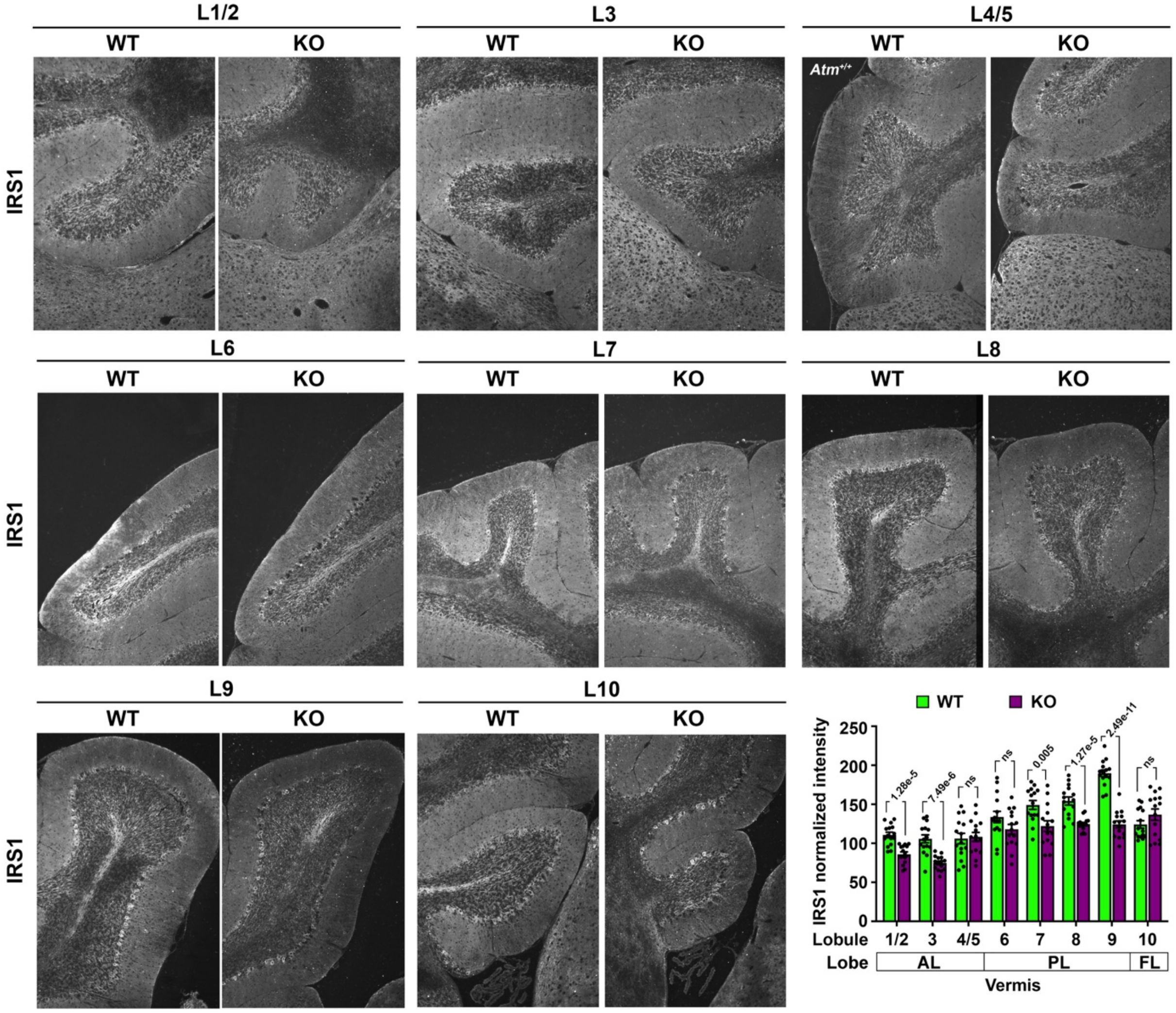

Supplementary Fig.10. Representative immunohistology images of sagittal sections dissecting the cerebellar vermis, illustrating intricate alterations in IRS1 signaling within Purkinje cells across various lobes in *Atm*-KO. Quantification of normalized intensities is depicted on the right (n=15, two-tailed unpaired t-test). N presents biological replicates. Values represent the mean ± s.d. Source data are provided as a Source Data file.

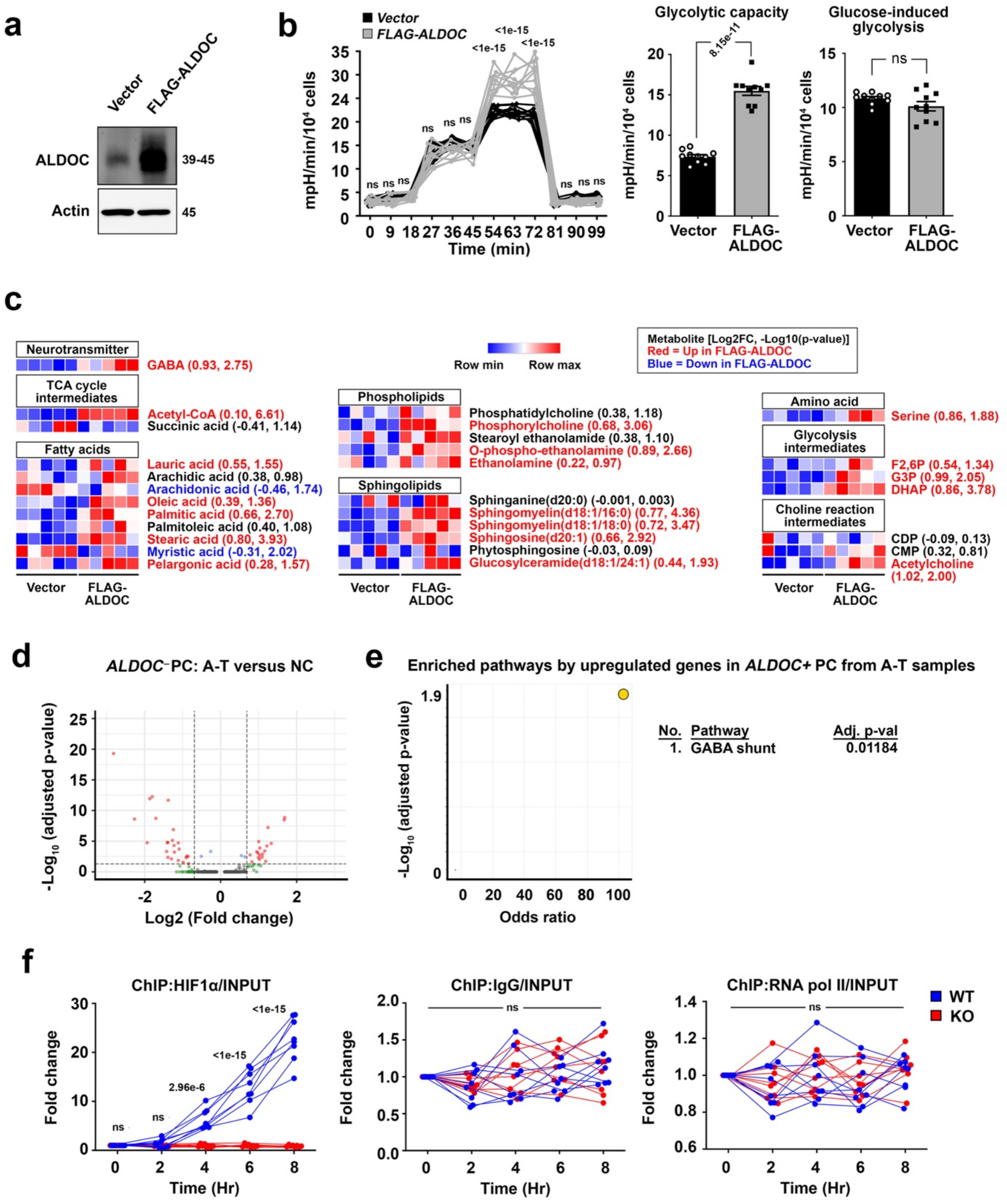

Supplementary Fig.11. Investigation of ALDOC-associated dysregulation and alterations in ATM deficiency. a, Representative immunoblot showcasing ALDOC levels in HT-22 cells after conducting the transfection procedure for 72 hours (n=6). **b,** Evaluation of glucose-triggered glycolytic rates through Extracellular Acidification Rate (ECAR) analysis conducted in HT-22 cells (n=10, two-way ANOVA). Histograms on the right represent glycolytic capacity and glucose-

induce glycolysis (n=10, two-tailed unpaired t-test). **c,** Heatmaps illustrating alterations in targeted lipid-related metabolite analyses in harvested HT-22 cells (n=5, two-tailed unpaired t-test). **d-e,** With reference to the publicly available single-nucleus transcriptomic data (SCP1300), **d,** volcano plot depicting alterations in gene expression levels of HIF1α targets according to the ChEA database in *ALDOC*- Purkinje cells of human A-T versus NC cerebellar samples (Wilcoxon rank-sum test with Bonferroni correction). **e,** Pathway enrichment analysis of down-regulated DEGs in *ALDOC*- Purkinje cells in A-T samples conducted on Enrichr[176] (Fisher exact test with correction). **f,** Quantification of ChIP-qPCR results illustrate the levels of HIF1α bound to its consensus binding sites predicted within the *Aldoc* promoter in the acute *ex vivo* cerebellar culture setting (n=8, two-way ANOVA). N presents biological replicates. Values represent the mean ± s.d. Source data are provided as a Source Data file.

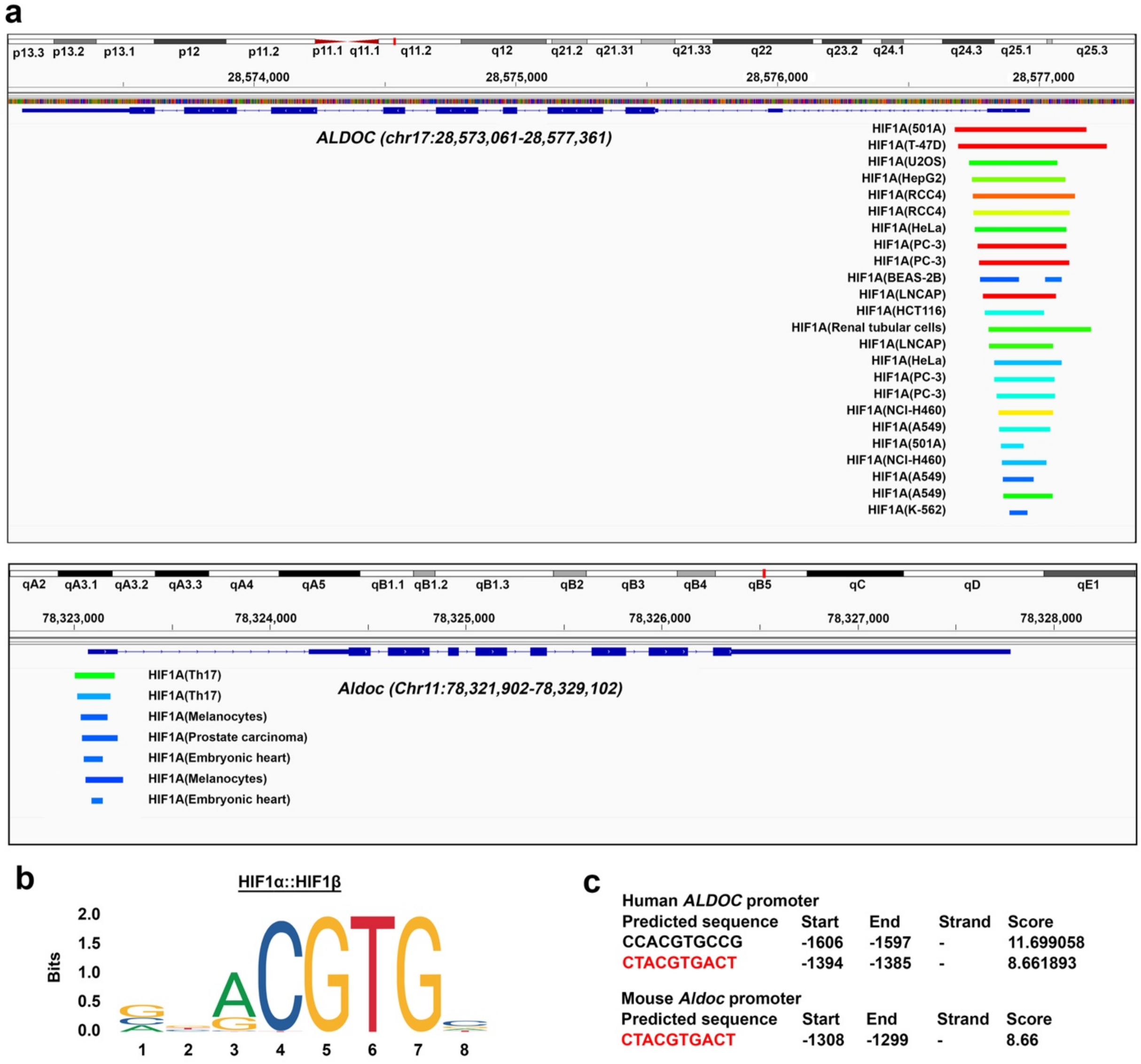

Supplementary Fig.12. ***ALDOC* is a potential target gene regulated by HIF1α. a,** Analysis of the human and mouse *ALDOC* gene promoters for HIF1α binding using ChIP-Atlas. **b,** Diagram illustrates the consensus binding motif for mouse HIF1α::HIF1β extracted from the JASPAR database. **c**, Anticipated HIF1α binding motifs within the promoters of the human and mouse *ALDOC* genes. The consensus site is highlighted in red.

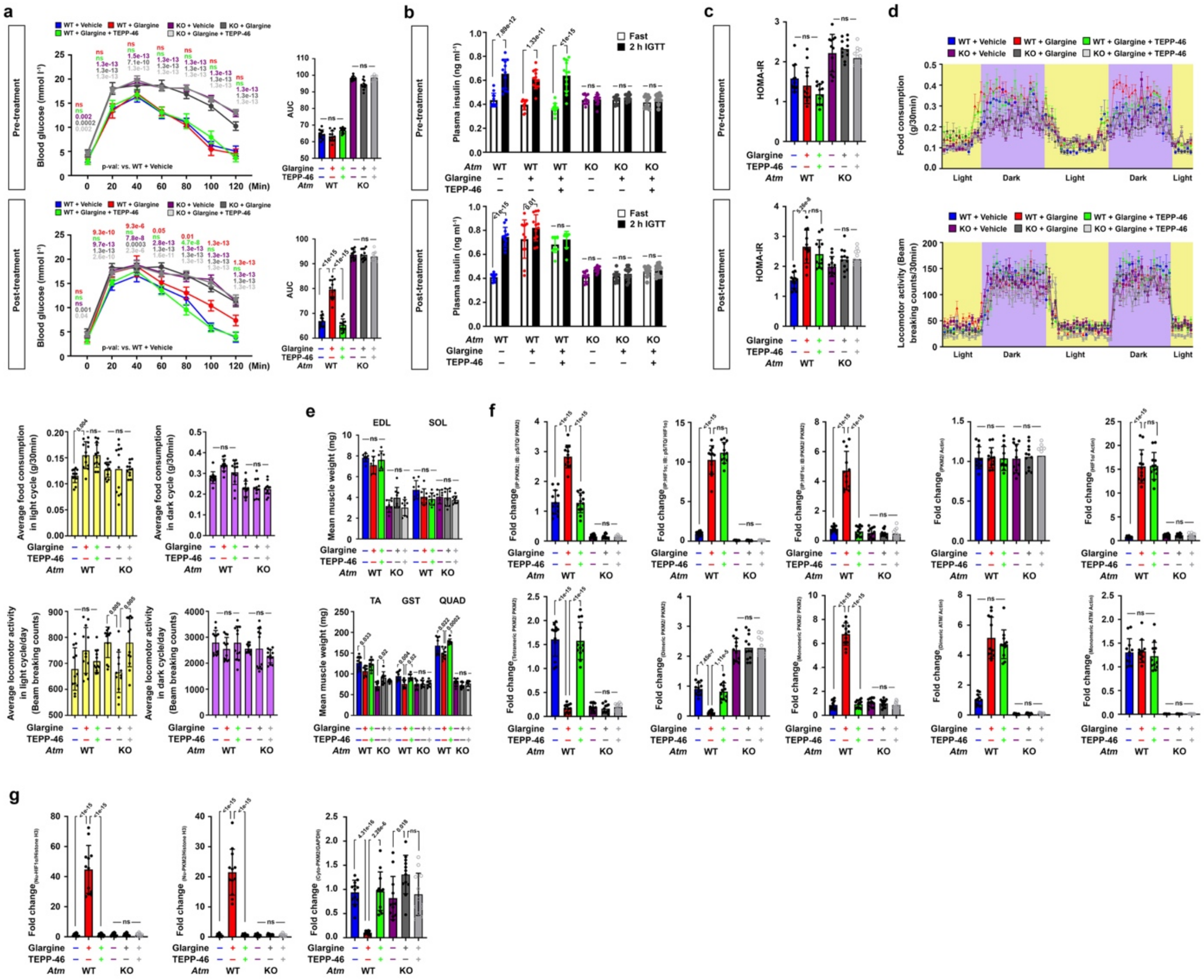

Supplementary Fig.13. Supplementary data corroborating comprehensive metabolic profiling in the context of hyperinsulinemia. a, Left: outcomes of intraperitoneal glucose tolerance tests (IGTTs) conducted before (Top) and after (Bottom) the treatment regimen as illustrated in Fig.5a (n=12, two-way ANOVA). Right: area under the curves (AUCs) (*n*=12, one-way ANOVA). **b**, Plasma insulin concentration levels were assessed during fasting (pre, top figure) and 2 hours following the IGTT (post, bottom figure) in mice as depicted in Fig.5a (n=12; two-way ANOVA). **c,** Overview of the HOMA-IR index in mice pre (Top) and post (Bottom) the treatment regimen (n=12; one-way ANOVA). **d,** Temporal variations in indirect calorimetry readings revealing alterations in both food intake and locomotor behavior. Additionally, an overview of the differences in food consumption and locomotor activities during light versus dark cycles is provided (n=12; one-way ANOVA for light cycle and Kruskal-Wallis test was used for dark cycle data analyses). **e,** Synopsis of the wet weights of major skeletal muscles collected following the treatment protocol (n=8, one-way ANOVA). Key muscles included are the EDL (extensor digitorum longus), SOL (soleus), TA (tibialis anterior), GST (gastrocnemius), and QUAD (quadriceps). **f-g,** Quantitative analysis of the blot intensities displayed in Fig.5g-h (n=12; one-way ANOVA for all except for "Reduced ATM (Dimer)/Actin" where Kruskal-Wallis test was used). N presents biological replicates. Values represent the mean ± s.d. Source data are provided as a Source Data file.

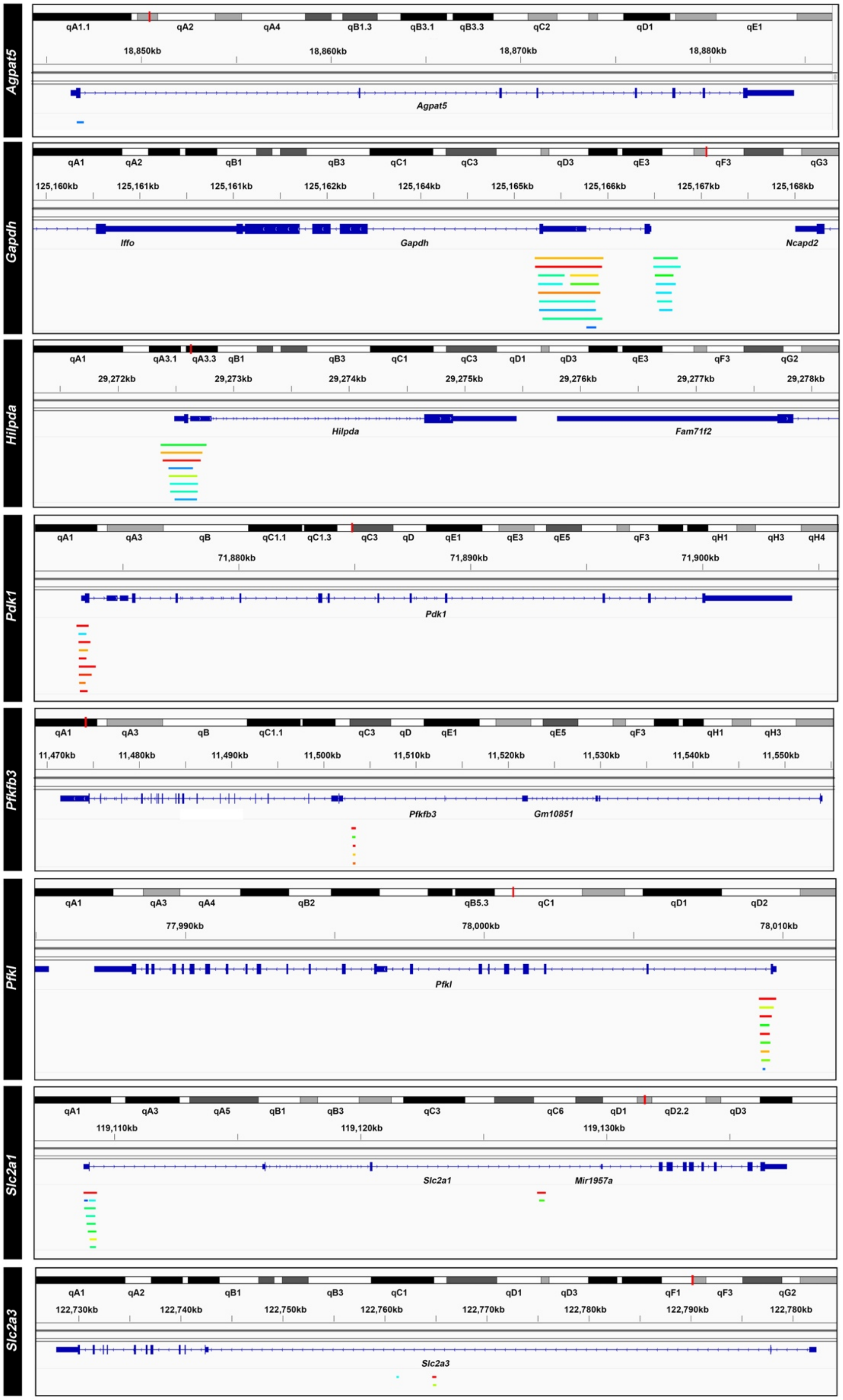

Supplementary Fig.14. Promoter analysis of glycolytic genes (Fig.5j) for HIF1α binding using the ChIP-Atlas database.

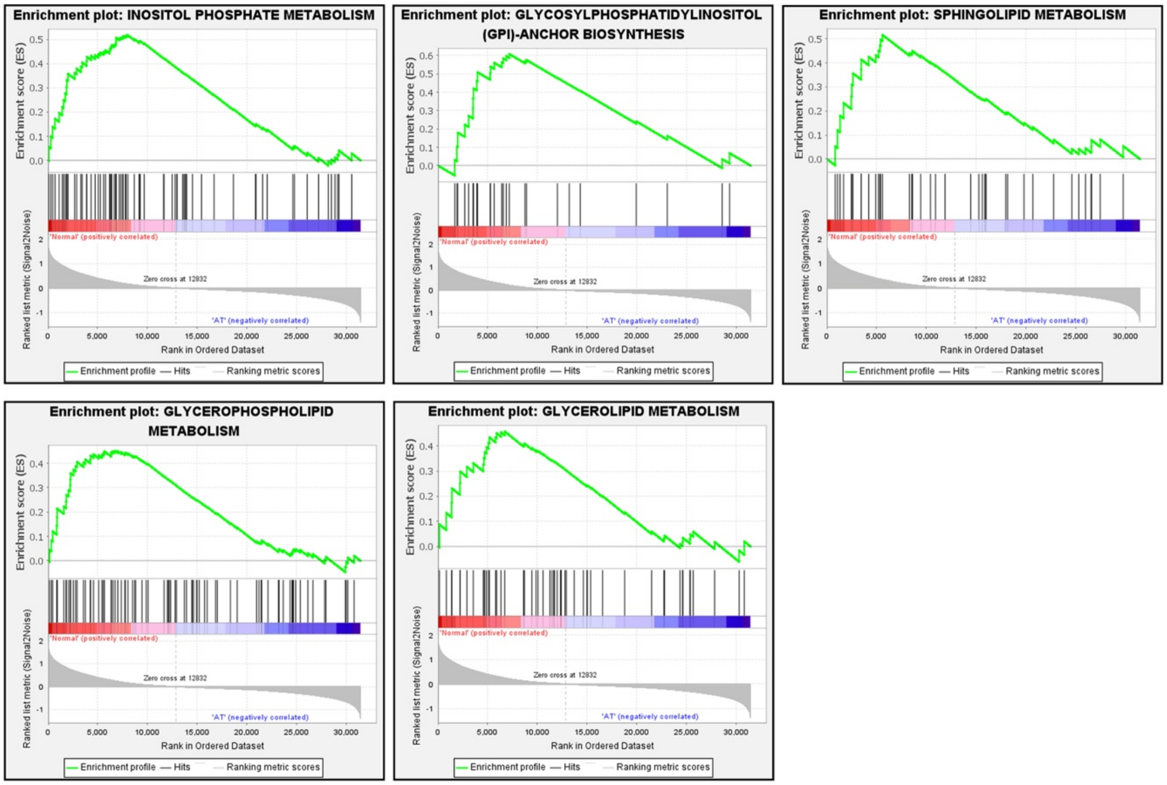

Supplementary Fig.15. Analysis of gene set enrichment using GSEA for markedly altered genes in human cerebellar samples from A-T patients (GSE61019). Prominent variances included pathways related to inositol phosphate metabolism (Nominal p-value = 0.000), glycosylphosphatidylinositol (GPI)-anchor biosynthesis (Nominal p-value = 0.008), sphingolipid metabolism (Nominal p-value = 0.0009), glycerophospholipid metabolism (Nominal p-value = 0.007), and glycerolipid metabolism (Nominal p-value = 0.033).

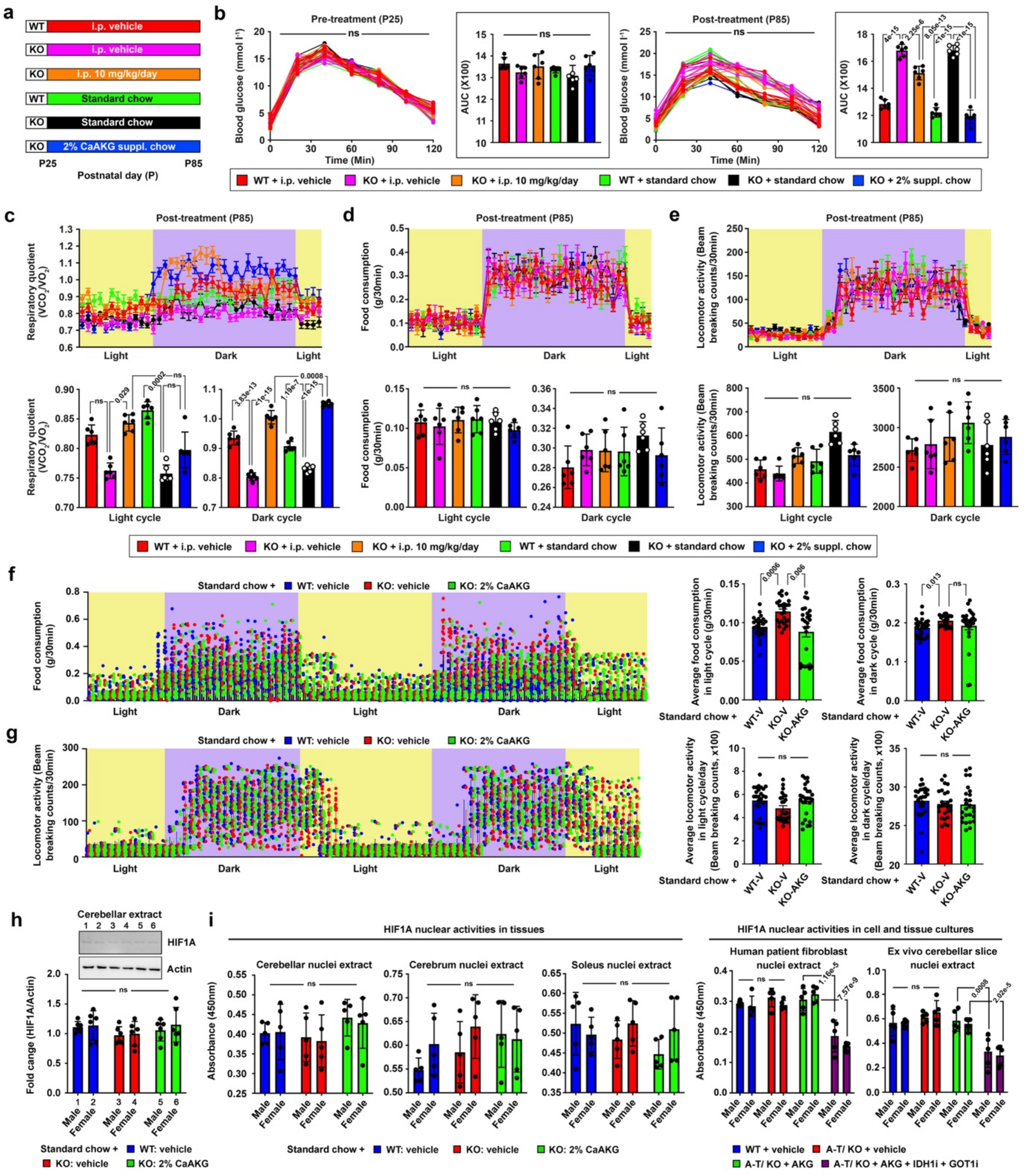

Supplementary Fig.16. Supplementary information regarding indirect calorimetry results from mice subjected to the CaAKG treatment regimen. a, Schematics of a 2-month long (~60 days) dietary regimen involving CaAKG supplementation (2% w/w) or vehicle alongside a standard laboratory diet, or an intraperitoneal regimen of 10 mg/kg/day CaAKG in newly weaned mice on postnatal day 25. **b,** Left: outcomes of intraperitoneal glucose tolerance tests (IGTTs) conducted before and after the treatment regimen as illustrated in Supplementary Fig.16a (n=6, two-way ANOVA). Right: area under the curves (AUCs) (n=6, Kruskal-Wallis test for pre-treatment and one-way ANOVA for post-treatment). **c-e,** Temporal variations in indirect

calorimetry readings showing alterations in **c,** respiratory quotient, **d,** food consumption and **e,** locomotor activities. A summary of the changes in these parameters during light versus dark cycles is provided (n=6, one-way ANOVA for all except that Kruskal-Wallis test was used for "RQ-Light cycle" and "Locomotor Activities-Light cycle"). **f-g,** Temporal variations in indirect calorimetry readings acquired after the entire treatment paradigm, showing alternations in **f,** food consumption and **g,** locomotor activity. On the right of each panel, a summary of the changes in of all these parameters during light versus dark cycles is provided (n=26, one-way ANOVA for locomotor activities and Kruskal-Wallis test for food consumption analyses). **h,** Representative immunoblot reflecting HIF1α levels remain unchanged after the treatment paradigm. Quantification of band intensities is shown at the bottom (n=12, one-way ANOVA). **i,** Measurement of HIF1α nuclear activities using HRE binding as a surrogate marker in nuclei extract of various insulin-sensitive tissues, including the cerebellum, cerebrum and soleus muscles harvested from mice subjected to treatment (n=10, one-way ANOVA) or human fibroblast cell and *ex vivo* cerebellar slice tissue culture samples (n=10, one-way ANOVA). N presents biological replicates. Values represent the mean ± s.d. Source data are provided as a Source Data file.

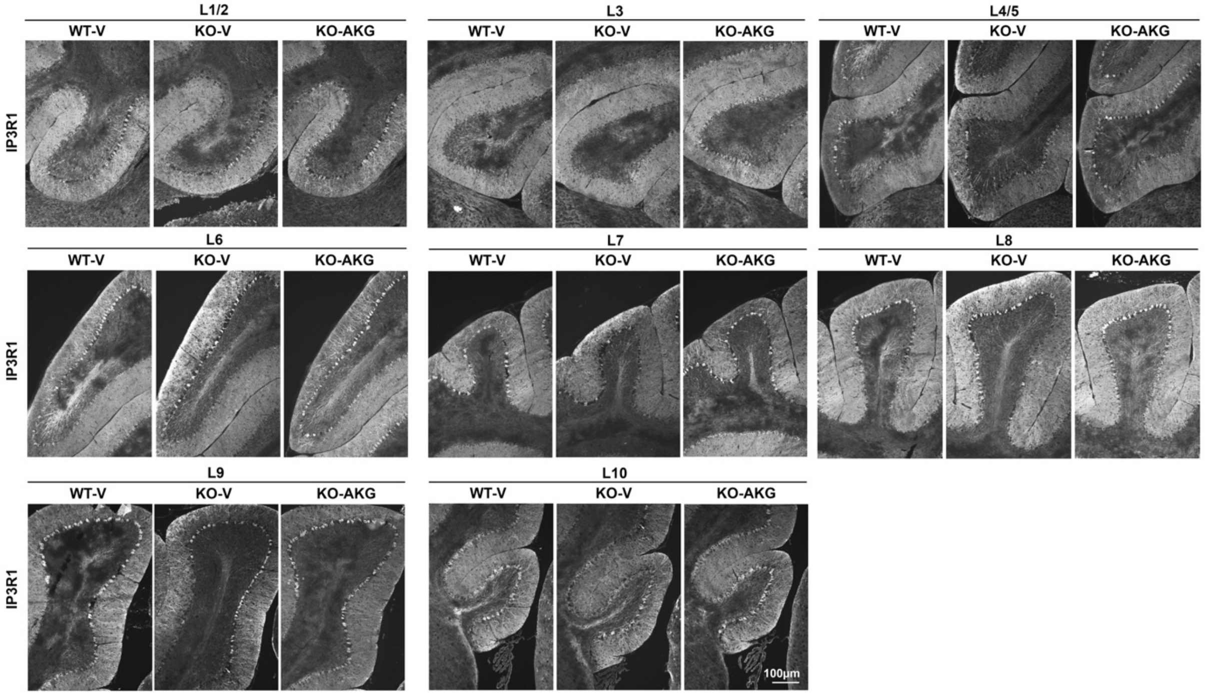

Supplementary Fig.17. Representative immunohistology images of sagittal sections dissecting the cerebellar vermis illustrating changes in the number of IP3R1+ Purkinje cells across various lobes in *Atm*-KO mice.

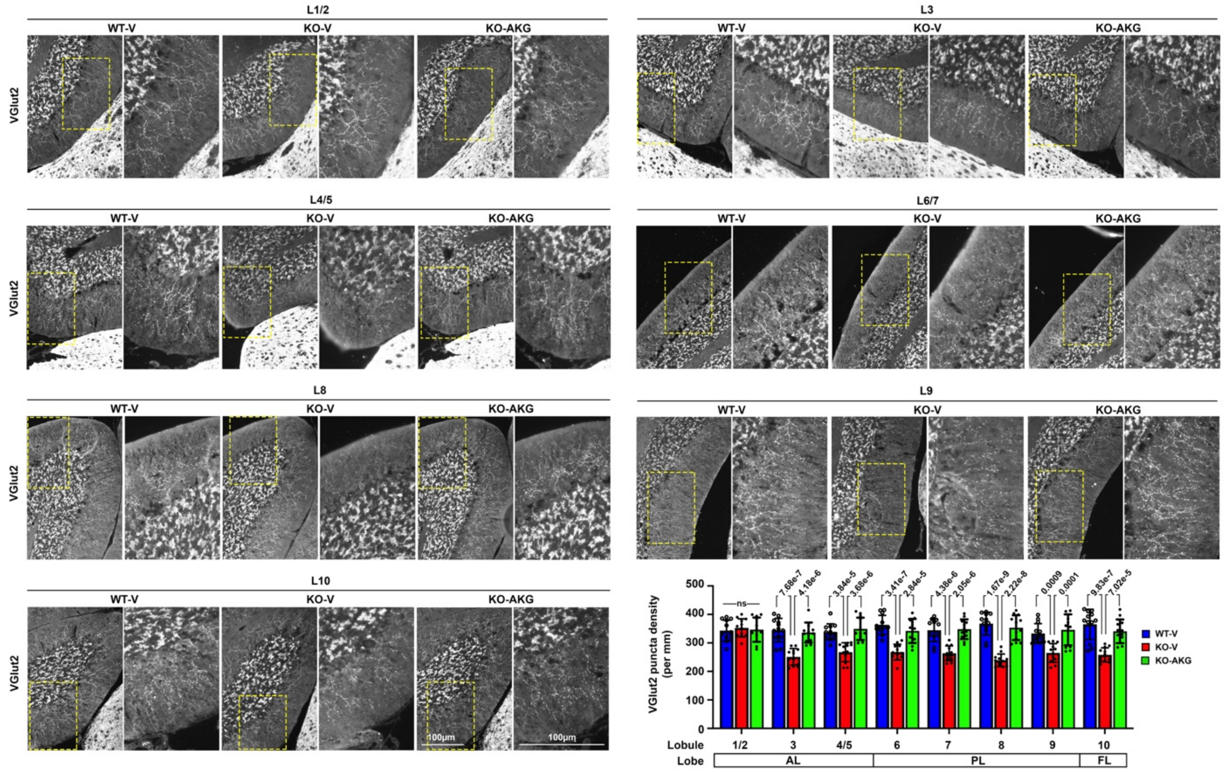

Supplementary Fig.18. Representative immunohistology images of sagittal sections dissecting the cerebellar vermis illustrating changes in the network of VGlut2 climbing fibers across various lobes in *Atm*-KO mice. Quantification of VGlut2 puncta density is shown (n=12, one-way ANOVA). N presents biological replicates. Values represent the mean ± s.d. Source data are provided as a Source Data file.

Supplementary Table 1. Special reagents list

**Antibodies**						
**Antibodies**	**Company**	**Catalog number**	**Size (kDa)**	**Application 1**	**Application 2**	**RRID**
Actin	Cell signaling Technology	#4967	45	Immunoblotting 1:1000		RRID:AB_330288
ALDOC	Thermo Fisher Scientific	PA5-27659	39	Immunoblotting 1:1000	Immunohistochemistry 1:100	RRID:AB_2545135
ATM	Cell signaling Technology	#2873	350	Immunoblotting 1:1000		RRID:AB_2062659
ATR	Cell signaling Technology	#2790	300	Immunoblotting 1:1000		RRID:AB_2227860
c-Myc	Cell signaling Technology	#5605	57-65	Immunoblotting 1:1000		RRID:AB_1903938
c-Myc	Abcam	ab32072	57-65	Chromatin immunoprecipitation 20ul		RRID:AB_731658
Calbindin	Cell signaling Technology	#2173	28	Immunoblotting 1:1000		RRID:AB_2183553
DNA-PKcs	Cell signaling Technology	#4602	450	Immunoblotting 1:1000		RRID:AB_10692482
GAD67	Thermo Fisher Scientific	PA5-21397	67	Immunoblotting 1:1000		RRID:AB_11153284
GAPDH	Cell signaling Technology	# 2118	37	Immunoblotting 1:1000		RRID:AB_561053
HIF1a	Cell signaling Technology	#14179	120	Chromatin immunoprecipitation 20ul	Co-immunoprecipitation 20ul	RRID:AB_2622225
Histone H3	Cell signaling Technology	#14269	17	Immunoblotting 1:1000		RRID:AB_2756816
Importin a3/ KPNA4	Novus Biologicals	NBP1-31260	60	Immunoblotting 1:1000	Co-immunoprecipitation 20ul	RRID:AB_2133841
INSRA	Cell signaling	#74118	135,220	Immunohistochemistry 1:100		RRID:AB_2799850

	Technology					
INSRB	Cell signaling Technology	#3025	95	Immunoblotting 1:1000	Immunohistochemistry 1:100	RRID:AB_2280448
IP3R1	Thermo Fisher Scientific	PA1-901	260	Immunohistochemistry 1:100		RRID:AB_2129984
IRS1	Cell signaling Technology	#2382	160	Immunoblotting 1:1000	Immunohistochemistry 1:100	RRID:AB_330333
IRS2	Cell signaling Technology	#4502	185	Immunohistochemistry 1:100		RRID:AB_2125774
NeuN	Thermo Fisher Scientific	# 702022	46,55	Immunoblotting 1:1000		RRID:AB_2633050
NOX1	Novus Biologicals	NBP1-31546	60,65	Immunoblotting 1:1000		RRID:AB_2235245
NOX2	Abcam	ab129068	60,65	Immunoblotting 1:1000		RRID:AB_11144496
NOX3	Thermo Fisher Scientific	PA5-38036	75	Immunoblotting 1:1000		RRID:AB_2554640
NOX4	Thermo Fisher Scientific	MA5-32090	60,65	Immunoblotting 1:1000		RRID:AB_2809383
pATM(S1981)	Abcam	ab36810	350	Immunoblotting 1:1000		RRID:AB_725573
Phospho-ATM/ATR substrate motif (pS/pT)Q	Cell signaling Technology	#6966	N/A	Immunoblotting 1:1000		RRID:AB_10949894
pINSRB(Tyr1345/Tyr1335)	Cell signaling Technology	#3026	95	Immunoblotting 1:1000		RRID:AB_2127116
pINSRB(Tyr1361)	Cell signaling Technology	#3023	95	Immunoblotting 1:1000		RRID:AB_2249189
pIRS1(Ser616/Ser612)	Thermo Fisher Scientific	44-550G	160	Immunoblotting 1:1000		RRID:AB_2533678
pIRS1(Ser794)	Thermo Fisher Scientific	PA5-36752	160	Immunoblotting 1:1000		RRID:AB_2553704
pIRS1(Tyr612/Tyr608)	Merck	09-432	160	Immunoblotting 1:1000		RRID:AB_1163457
PKM2	Cell signaling	#3198	60	Immunoblotting 1:1000	Co-immunopre	RRID:AB_2252325

	Technology				cipitation 20ul	
Rabbit IgG	Thermo Fisher Scientific	#02-6102	N/A	Co-immunoprecipitation 20ul	RNA-immunoprecipitation 20ul	RRID:AB_2532938
RNA pol II	Thermo Fisher Scientific	#39097	250	Chromatin immunoprecipitation 20ul		RRID:AB_2732926
VGAT	Thermo Fisher Scientific	PA5-27569	50	Immunoblotting 1:1000		RRID:AB_2545045
VGlut2	Thermo Fisher Scientific	MA5-27613	60	Immunohistochemistry 1:100		RRID:AB_2735411

**Special chemical/Kits**		
**Special chemicals/kits**	**Company**	**Catalog number**
1,2,3,4-$^{13}C_4$-α-KG	Cambridge Isotope	6363-53-7
$^{13}C_5$-U-glutamine	Sigma-Aldrich	605166
$^{13}C_6$-U-glucose	Cambridge Isotope	CLM-1396
Accu-Chek Glucose Meter and testing strips	Roche	N/A
Ammonia Assay Kit	Abcam	ab83360
Borosilicate glass	Sutter Instrument Company	B100-75-10
CellROX Green Reagent, for oxidative stress detection	Thermo Fisher Scientific	C10444
GLX7013114	Smolecule	S529008
HIF-1 alpha Transcription Factor Assay Kit	Abcam	ab133104
High-Capacity cDNA Reverse Transcription kit	Applied Biosystems	4368814
KU 60019	Calbiochem	5319780001
L-Arginine-HCl, 13C6, 15N4 for SILAC	Thermo Fisher	89990
L-Lysine-2HCl, 13C6, 15N2 for SILAC	Thermo Fisher	88209
Lipofectamine 2000	Thermo Fisher Scientific	11668019
Lipofectamine LTX with plus reagent	Thermo Fisher Scientific	15338100
Magna RIP™ RNA-binding protein immunoprecipitation kit	Millipore	17-700
Mito Fuel Flex Tests	Agilent	103260-100
NuPAGE™ 10%, Bis-Tris, 1.0–1.5 mm, Mini Protein Gels	Thermo Fisher Scientific	NP0302BOX
Pierce™ IP lysis buffer	Thermo Fisher Scientific	PI87787
Pyruvate kinase assay	Abcam	ab83432
Serum Triglyceride Determination Kit	Sigma-Aldrich	TR0100
TEPP-46 (ML265)	Selleckchem	S7302
Titansphere Phos-TiO beads and columns	GL Science Inc	5010-21307

Ultra Sensitive Mouse Insulin ELISA Kit	Crystal Chem	90080
XF Glycolysis Stress Test	Agilent	103020-100
**Software tools**		
**Name**	**Website**	
ChIP-Atlas	https://chip-atlas.org/	
Enrich R	https://maayanlab.cloud/Enrichr/	
Gene Set Enrichment Analysis (GSEA)	https://www.gsea-msigdb.org/gsea/index.jsp	
GeneVenn	https://www.bioinformatics.org/gvenn/	
GEO2R	https://www.ncbi.nlm.nih.gov/geo/geo2r/	
HADDOCK2.4	https://wenmr.science.uu.nl/haddock2.4/	
Human Brain Transcriptome database	https://hbatlas.org	
IGV genome browser	https://software.broadinstitute.org/software/igv/	
JASPAR 2022 database	https://jaspar.genereg.net/	
MaxQuant	https://www.maxquant.org/	
MetaboAnalyst	https://www.metaboanalyst.ca/MetaboAnalyst/ModuleView.xhtml	
Metascape	https://metascape.org/gp/index.html#/main/step1	
Morpheus	https://software.broadinstitute.org/morpheus/	
NCBI Gene	https://www.ncbi.nlm.nih.gov/gene/	
NCBI Genome Data Viewer	https://www.ncbi.nlm.nih.gov/genome/gdv/	
Phospho-Analyst	https://analyst-suites.org/apps/phospho-analyst/	
pLogo	https://plogo.uconn.edu/	
PyMOL 2.5	https://pymol.org/2/	
R Studio	https://posit.co/download/rstudio-desktop/	
SWISS-MODEL	https://swissmodel.expasy.org/	
STRING	https://string-db.org/cgi/input?sessionId=bqvVKq63Bicf&input_page_show_search=off	
ChIP-Atlas	https://chip-atlas.org/	

**Chromatin immunoprecipitation primers**					
**Gene of interest**	**GenBank Accession**	**Forward primer (5'->3')**	**Reverse primer (5'->3')**	**Amplicon Size (bp)**	**Annealing temperature (oC)**
Aldoc	NC_000077.7	AAGTGGGGCACTGTTAGGTGG	GACGCGAGCATCTCAGGCTC	494	67.5
GLS2	NC_000012.12	CTTCTAGATAATGTCCTATGTACTGACA	TTAGTCCCCTGACTCTCACTTTG	499	62.7
**RNA immunoprecipitation primer**					
**Gene of interest**	**GenBank Accession**	**Forward primer (5'->3')**	**Reverse primer (5'->3')**	**Amplicon Size (bp)**	**Annealing temperature (oC)**
C-MYC	NM_001354870.1	TAATCTCCGCCCACCGGCC	GCTTACCTGGTTTTCCACTACCCGAAA	610	69.5
**Quantitative PCR primers**					
**Gene of interest**	**GenBank Accession**	**Forward primer (5'->3')**	**Reverse primer (5'->3')**	**Amplicon Size (bp)**	**Annealing temperature (oC)**
Agpat5	NM_026792	CACACGTACTCTATGCGCTAC	AAGAAGAGCACCATGTTCTGG	173	63.3

Gapdh	NM_008084	AGGTCGGTGTGAACGGATTTG	TGTAGACCATGTAGTTGAGGTCA	123	63.8
Hilpda	NM_001190461	TGCTGGGCATCATGTTGACC	TGACCCCTCGTGATCCAGG	109	66.2
Lpin3	NM_001199118	CAAACCTCGTGGTGAAAATCAAC	CCACAGTGCTCTCAGGTAAGT	108	61
Pdk1	NM_172665	GGACTTCGGGTCAGTGAATGC	TCCTGAGAAGATTGTCGGGGA	122	65.6
Pfkbp3	NM_001177758	CAACTCCCCAACCGTGATTGT	GAGGTAGCGAGTCAGCTTCTT	82	64.5
Pfkl	NM_008826	GGAGGCGAGAACATCAAGCC	CGGCCTTCCCTCGTAGTGA	113	66
Rpl19	NM_001159483	ATGAGTATGCTCAGGCTACAGA	GCATTGGCGATTTCATTGGTC	104	63.6
Slc2a1	NM_011400	CAGTTCGGCTATAACACTGGTG	GCCCCCGACAGAGAAGATG	156	63.9
Slc2a3	NM_011401	ATGGGGACAACGAAGGTGAC	GTCTCAGGTGCATTGATGACTC	107	63.9

Plasmids

**Name**	**Company**	**Catalog number**
AAV-Synapsin-mCherry-C1-WPRE	Addgene	159956
Cyto-roGFP	Addgene	49345
pEGFP-C1-PKM2	Addgene	64698
pg-HIF-1alpha-EGFP	Addgene	87204
pGFP-C-shLenti shRNA Vector	Origene	TR30023
pGFP-C-shLenti-Atm (Lentiviral particle)	Origene	TL500154V
pGFP-C-shLenti-Atr (Lentiviral particle)	Origene	TL519184V
pGFP-C-shLenti-Nox4 (Lentiviral particle)	Origene	TL512359
pGFP-C-shLenti-Prkdc/DNA-PK (Lentiviral particle)	Origene	TL512107V
pReceiver-Lv241-Aldoc (C-Flag-SV40-Puromycin)	GeneCopoeia	ULP-Mm01162-Lv241-100
