# Supplementary material for: Alpha-ketoglutarate mitigates insulin resistance and metabolic inflexibility in a mouse model of Ataxia-Telangiectasia
Source: Nat Commun. 2025 Oct 21;16:9312. doi: 10.1038/s41467-025-64360-8 (PMC12540671; doi:10.1038/s41467-025-64360-8)
Supplement: Supplementary file 2 — Description of Additional Supplementary Files [file 41467_2025_64360_MOESM2_ESM.pdf]

Description of Additional Supplementary Files

Supplementary Dataset 1: Summary of the list of phosphopeptides reproducibly found in quantitative label-free phosphoproteomics analysis. Treatment group labelling: UWT = "WT – Glargine"; SWT = "WT + Glargine"; UKO = "KO – Glargine" and SKO = "KO + Glargine".

Supplementary Dataset 2: Summary of the list of total peptides reproducibly found in quantitative label-free phosphoproteomics analysis. Treatment group labelling: UWT = "WT – Glargine"; SWT = "WT + Glargine"; UKO = "KO – Glargine" and SKO = "KO + Glargine".

Supplementary Dataset 3: Summary of Bulk RNA-Seq of cerebellar tissues harvested from 6-month-old mice.
