# Supplementary material for: Alpha-ketoglutarate mitigates insulin resistance and metabolic inflexibility in a mouse model of Ataxia-Telangiectasia
Source: Nat Commun. 2025 Oct 21;16:9312. doi: 10.1038/s41467-025-64360-8 (PMC12540671; doi:10.1038/s41467-025-64360-8)
Supplement: Supplementary file 6 — Reporting Summary [file 41467_2025_64360_MOESM6_ESM.pdf]

nature portfolio

Corresponding author(s): Kim Hei-Man CHOW

Last updated by author(s): 2025/09/10

Reporting Summary

Nature Portfolio wishes to improve the reproducibility of the work that we publish. This form provides structure for consistency and transparency in reporting. For further information on Nature Portfolio policies, see our Editorial Policies and the Editorial Policy Checklist.

Statistics

For all statistical analyses, confirm that the following items are present in the figure legend, table legend, main text, or Methods section.

n/a	Confirmed	
☐	☒	The exact sample size (*n*) for each experimental group/condition, given as a discrete number and unit of measurement
☐	☒	A statement on whether measurements were taken from distinct samples or whether the same sample was measured repeatedly
☐	☒	The statistical test(s) used AND whether they are one- or two-sided<br>*Only common tests should be described solely by name; describe more complex techniques in the Methods section.*
☒	☐	A description of all covariates tested
☐	☒	A description of any assumptions or corrections, such as tests of normality and adjustment for multiple comparisons
☐	☒	A full description of the statistical parameters including central tendency (e.g. means) or other basic estimates (e.g. regression coefficient) AND variation (e.g. standard deviation) or associated estimates of uncertainty (e.g. confidence intervals)
☐	☒	For null hypothesis testing, the test statistic (e.g. *F*, *t*, *r*) with confidence intervals, effect sizes, degrees of freedom and *P* value noted<br>*Give P values as exact values whenever suitable.*
☒	☐	For Bayesian analysis, information on the choice of priors and Markov chain Monte Carlo settings
☒	☐	For hierarchical and complex designs, identification of the appropriate level for tests and full reporting of outcomes
☐	☒	Estimates of effect sizes (e.g. Cohen's *d*, Pearson's *r*), indicating how they were calculated

Our web collection on statistics for biologists contains articles on many of the points above.

Software and code

Policy information about availability of computer code

Data collection	The following commercial software was used to collect data:<br>Magellan (TECAN plate reader); LAS X (Leica); Seahorse Wave Desktop software (Agilent); LightCycler 480 Software (Roche); GenePix Pro6.0 (Molecular Devise); Agilent MassHunter software (Agilent); Freeze Frame Software; Freeze Frame software (Actimetrics); Smart 3.0 video tracking system (Panlab, Harvard Apparatus); AB Sciex Analyst 1.4.2 (Applied Biosystems); Xcalibur Software 2.2 (Thermo Fisher Scientific); Metabolism software (Pan Lab/Harvard Instruments); MASCOT 2.3 software (Matrix Science), HiSeq X Ten platform (Illumina), NIS-Elements software (Nikon), LAS X Life Science Microscope software (Leica).
Data analysis	The following commercial software was used to analyze data:<br>Microsoft Excel; Morpheus platform (Broad Institute); BBrowser2.7.48 (BioTuring Inc); (GEO2R (NIH); Prism 10 (Graphpad); Seahorse Wave Desktop software (Agilent); Metabolite Set Enrichment Analysis (Metaboanalyst); Gene Set Enrichment Analysis (Broad Institute); Agilent OpenLab CDS (ChemStation Edition); Freeze Frame software (Actimetrics); Smart 3.0 video tracking system (Panlab, Harvard Apparatus); Image J v1.8.0 (NIH); AB Sciex Analyst 1.4.2 (Applied Biosystems); Xcalibur Software 2.2 (Thermo Fisher Scientific), Metabolism software (Pan Lab/Harvard Instruments), LAS X Life Science Microscope software (Leica), MASCOT 2.3 software (Matrix Science), SWISS-MODEL algorithm (Swiss Institute of Bioinformatics), PyMOL software (DeLano Scientific), HADDOCK 2.4 (BioExcel Center of Excellence for Biomolecular Research), ChIP-Atlas (Kyoto University), Integrative Genomics Viewer (Broad Institute), JASPAR 2022 database (Elixir Norway, University of Copenhagen, Centre for Molecular Medicine and Therapeutics, London Institute of Medical Sciences and Centre for Molecular Medicine Norway), R studio (Posit Software), Enrichr (Icahn School of Medicine at Mount Sinai), FastQC V. 0.11.9 (Babraham Bioinformatics), STAR software (MIT), Ingenuity IPA (Qiagen), MaxQuant (Max Planck Institute), Phospho-Analyst (Monash).

For manuscripts utilizing custom algorithms or software that are central to the research but not yet described in published literature, software must be made available to editors and reviewers. We strongly encourage code deposition in a community repository (e.g. GitHub). See the Nature Portfolio guidelines for submitting code & software for further information.

Data

Policy information about availability of data

All manuscripts must include a data availability statement. This statement should provide the following information, where applicable:

- Accession codes, unique identifiers, or web links for publicly available datasets
- A description of any restrictions on data availability
- For clinical datasets or third party data, please ensure that the statement adheres to our policy

The single-nucleus RNA-sequencing dataset obtained from the human cerebellum in Ataxia Telangiectasia can be found under the accession code SCP1300 from the Single Cell Portal provided by The Broad Institute [https://singlecell.broadinstitute.org/single_cell/study/SCP1300/single-cell-atlas-of-the-human-cerebellum-in-ataxia-telangiectasia] (PMID: 38159274). The snRNA-seq of the mouse cerebellar cortex can be found under the accession code SCP795 from the Single Cell Portal [https://singlecell.broadinstitute.org/single_cell/study/SCP795/a-transcriptomic-atlas-of-the-mouse-cerebellum] (PMID: 34616064). The cerebellar cortex expression in ataxia-telangiectasia patients and normal controls from expression profiling by array can be found in GEO Omnibus under the accession code GSE61019 [https://www.ncbi.nlm.nih.gov/geo/query/acc.cgi?acc=GSE61019] (PMID: 26510954). The bulk RNA-seq data generated in this study have been deposited in GEO Omnibus under the accession code GSE222655 [https://www.ncbi.nlm.nih.gov/geo/query/acc.cgi?acc=GSE222655]. The phospho-proteomics/ proteomics data have been deposited in PRIDE database under the accession code PXD062018 [https://www.ebi.ac.uk/pride/archive/projects/PXD062018]. The metabolomics/ lipidomics raw data generated in this study have been included in Source data file. The processed "Minimum dataset" data that are necessary to interpret, verify and extend the research in the article, are located in "Supplementary Dataset" files". Source data are provided with this paper.

Note to Editor:
The data deposited on PRIDE is still in a private status as the database requires a Pubmed ID for publishing the data. The data can be accessed using the reviewer access, the token is 0zr0jBgpANOf. Alternatively, reviewer can access the dataset by logging in to the PRIDE website using the following account details:
Username: reviewer_pxd062018@ebi.ac.uk
Password: fYOFhvztiH0T

Research involving human participants, their data, or biological material

Policy information about studies with human participants or human data. See also policy information about sex, gender (identity/presentation), and sexual orientation and race, ethnicity and racism.

Reporting on sex and gender	Not applicable.
Reporting on race, ethnicity, or other socially relevant groupings	Not applicable.
Population characteristics	Not applicable.
Recruitment	Not applicable.
Ethics oversight	Not applicable.

Note that full information on the approval of the study protocol must also be provided in the manuscript.

Field-specific reporting

Please select the one below that is the best fit for your research. If you are not sure, read the appropriate sections before making your selection.

☒ Life sciences ☐ Behavioural & social sciences ☐ Ecological, evolutionary & environmental sciences

For a reference copy of the document with all sections, see nature.com/documents/nr-reporting-summary-flat.pdf

Life sciences study design

All studies must disclose on these points even when the disclosure is negative.

Sample size	Sample size was calculated based on previous data (e.g. Chow et al., Nature Neuroscience, 2019 and other recent publications) assuming a power of 80 & and an alpha value of 0.05.
Data exclusions	No data exclusion was performed.
Replication	Number of replications are noted in the figure legends. Replicate data reproduced the initial observations.
Randomization	Litter mate mice were randomly assigned to control or experimental groups following genotyping.
Blinding	Behavioral experiments were performed blinded and analyzed by different scientists. Blinding was not possible in each set of cell-based experiment as the same scientist performed the treatment and analyzes, however we had the replicating rounds of the same experiments

performed by different scientists to confirm reproducibility.

Reporting for specific materials, systems and methods

We require information from authors about some types of materials, experimental systems and methods used in many studies. Here, indicate whether each material, system or method listed is relevant to your study. If you are not sure if a list item applies to your research, read the appropriate section before selecting a response.

Materials & experimental systems

n/a	Involved in the study	
☐	☒	Antibodies
☐	☒	Eukaryotic cell lines
☒	☐	Palaeontology and archaeology
☐	☒	Animals and other organisms
☒	☐	Clinical data
☒	☐	Dual use research of concern
☒	☐	Plants

Methods

n/a	Involved in the study	
☒	☐	ChIP-seq
☒	☐	Flow cytometry
☒	☐	MRI-based neuroimaging

Antibodies

Antibodies used: The complete list of antibodies used in this study can be found in the Supplementary Table 1.

Validation: All antibodies are identified with their corresponding RRID code, as listed in the Supplementary Table 1. These antibodies are also used in multiple previously published studies from our lab and others.

Eukaryotic cell lines

Policy information about cell lines and Sex and Gender in Research

Cell line source(s): HEK293FT (ThermoFisher)

Authentication: The identity of the cell line was frequently checked by their morphological features and did not show any signs of cross-contamination. However they have not been authenticated by short tandem repeat (STR) profiling.

Mycoplasma contamination: The cell line was regularly tested in the laboratory for mycoplasma contamination and were mycoplasma free.

Commonly misidentified lines (See ICLAC register): No commonly misidentified cell lines were used in this study.

Animals and other research organisms

Policy information about studies involving animals; ARRIVE guidelines recommended for reporting animal research, and Sex and Gender in Research

Laboratory animals: B6;129S4-Atmtm1Bal/J (Atm +/+, Atm +/- or Atm -/-) mice were obtained from the Jackson Laboratory. These mice were maintained and bred in the Laboratory Animal Service Centre of the Chinese University of Hong Kong. Animals were housed under a 12 h light/dark cycle at room temperature (22±2 oC) and constant humidity levels at around 50-70%, with food and water provided ad libitum in a specific pathogen free (SPF) environment.

For all experiments, unless otherwise stated (see more details below on Reporting on sex), no inclusion or exclusion criteria were applied other than the animal's genotype, HOMA-IR status and age. The age of the animals used for each experiment is clearly stated in the corresponding figure legends or text. With these criteria, mice were randomly chosen among the available colonies.

Wild animals: No wild animals were used in the present study.

Reporting on sex: Whenever applicable, sex of the samples are indicated in the source data file, and all statistical analyses performed can be found in the source data files.

One exception arose in the context of the study involving streptozotocin (STZ) injection. It is noteworthy that STZ, a pharmacological agent utilized to induce diabetes in laboratory mice, as documented in the literature (Furman, 2015), exhibits differential sensitivity in female mice due to potential protective effects conferred by estrogen, as elucidated by Paik and colleagues (Paik et al., 1982). Consequently, owing to the inherent complexities introduced by the estrous cycle in fertile female mice and the disparate STZ dosages required to elicit pancreatic β-cell toxicity (with female subjects typically needing higher STZ doses than their male counterparts), the existing literature have favoured the utilization of male animals in STZ-induced diabetic mouse studies (Kolb, 1987, Furman, 2021). Considering these reasons, we declare that this set of experiments was conducted only with male subjects, to ensure consistency and comparability with existing literature and methodologies.

Furman, B.L. Streptozotocin-Induced Diabetic Models in Mice and Rats. Curr Protoc Pharmacol 70, 5 47 41-45 47 20 (2015).
Paik, S.G., Michelis, M.A., Kim, Y.T. & Shin, S. Induction of insulin-dependent diabetes by streptozotocin. Inhibition by estrogens and potentiation by androgens. Diabetes 31, 724-729 (1982).
Kolb, H. Mouse models of insulin dependent diabetes: low-dose streptozocin-induced diabetes and nonobese diabetic (NOD) mice. Diabetes Metab Rev 3, 751-778 (1987).
Furman, B.L. Streptozotocin-Induced Diabetic Models in Mice and Rats. Curr Protoc 1, e78 (2021).

Field-collected samples
No field-collected samples were used in the present study.

Ethics oversight
Mouse colonies were maintained and bred in the Laboratory Animal Services Centre of The Chinese University of Hong Kong (CUHK). All animal experimental protocols were approved by the Animal Ethics Committees at CUHK; and their care was in accord with the institutional and Hong Kong guidelines.

Note that full information on the approval of the study protocol must also be provided in the manuscript.

Plants

Seed stocks
Not applicable.

Novel plant genotypes
Not applicable.

Authentication
Not applicable.
